# The Dancing Brain: Structural and Functional Signatures of Expert Dance Training

**DOI:** 10.3389/fnhum.2017.00566

**Published:** 2017-11-27

**Authors:** Agnieszka Z. Burzynska, Karolina Finc, Brittany K. Taylor, Anya M. Knecht, Arthur F. Kramer

**Affiliations:** ^1^Department of Human Development and Family Studies, Molecular, Cellular and Integrative Neurosciences, Colorado State University, Fort Collins, CO, United States; ^2^Centre for Modern Interdisciplinary Technologies, Nicolaus Copernicus University in Toruń, Toruń, Poland; ^3^Center for Neurobehavioral Research, Boys Town National Research Hospital, Boys Town, NE, United States; ^4^The Beckman Institute for Advanced Science and Technology, University of Illinois at Urbana–Champaign, Urbana, IL, United States; ^5^Departments of Psychology and Mechanical and Industrial Engineering, Northeastern University, Boston, MA, United States

**Keywords:** dance, MRI and fMRI, MRI volumetry, DTI, cognition, training effects, functional connectivity (FC), motor skills

## Abstract

Dance – as a ritual, therapy, and leisure activity – has been known for thousands of years. Today, dance is increasingly used as therapy for cognitive and neurological disorders such as dementia and Parkinson’s disease. Surprisingly, the effects of dance training on the healthy young brain are not well understood despite the necessity of such information for planning successful clinical interventions. Therefore, this study examined actively performing, expert-level trained college students as a model of long-term exposure to dance training. To study the long-term effects of dance training on the human brain, we compared 20 young expert female Dancers with normal body mass index with 20 age- and education-matched Non-Dancers with respect to brain structure and function. We used diffusion tensor, morphometric, resting state and task-related functional MRI, a broad cognitive assessment, and objective measures of selected dance skill (Dance Central video game and a balance task). Dancers showed superior performance in the Dance Central video game and balance task, but showed no differences in cognitive abilities. We found little evidence for training-related differences in brain volume in Dancers. Dancers had lower anisotropy in the corticospinal tract. They also activated the *action observation network* (AON) to greater extent than Non-Dancers when viewing dance sequences. Dancers showed altered functional connectivity of the AON, and of the general motor learning network. These functional connectivity differences were related to dance skill and balance and training-induced structural characteristics. Our findings have the potential to inform future study designs aiming to monitor dance training-induced plasticity in clinical populations.

## Introduction

Dance is a pleasurable and captivating activity that involves motor, cognitive, visuospatial, social, and emotional engagement. Although practiced for thousands of years in rituals and as a leisure activity, the long-term effects of systematic dance training on cognition, and brain structure and function are not well understood.

Currently, there is increasing interest in dance as a therapeutic intervention for various clinical groups, ranging from developmental disorders such as Down syndrome (Lifshitz-Vahav et al., 2016), to neurological disorders such as schizophrenia (Martin et al., 2016) and mood disorder (depression; Meekums et al., 2015), neuromotor disorders such as Parkinson’s disease (McNeely et al., 2015), to dementia prevention and management (Ballesteros et al., 2015; Adam et al., 2016). Despite the popularity of dance as a cognitive or mental therapy, recent reviews question the effectiveness of short-term dance therapy in neurological disorders such as schizophrenia (Ren and Xia, 2013) and depression (Meekums et al., 2015), concluding there is no evidence for or against dance as a treatment. Conversely, epidemiological studies found that people with a life-long history of dancing were less likely to be diagnosed with dementia or to experience age-related cognitive decline (Verghese et al., 2003). Indeed, in a recent randomized clinical trial we have shown subtle yet promising benefits of a 6-month, 3-times per week dance intervention on the microstructure of the fornix in healthy older adults (Burzynska et al., 2017). Surprisingly, similar trials using neuroimaging to study the effects of dance in neurodegenerative disease patients are still in the early stages (e.g., Earhart et al., 2015).

Given the market value of dance therapy, and the opportunity that dance may become for our aging population, it is a timely question *whether* and *how* long-term dance training affects the healthy adult human brain and mind. As different types of dance activities at an amateur level may result in mixed effects, studying young expert dancers seems like a logical first step to understand the effects of long-term systematic training on the healthy human brain. Surprisingly, there have been few systematic attempts to understand expert dancers’ brains, often focusing only on a single aspect of brain structure or function with limited objective assessment of cognitive and dance ability (Karpati et al., 2015).

### Dance Expertise and Brain Structure

Hänggi et al. (2010) explored differences in brain structure between a sample of 10 professional ballet Dancers and 10 individuals with no professional dance training. The data indicated significantly *decreased* volume near the premotor and supplementary motor cortex, and in the putamen and corticospinal tract in Dancers (Hänggi et al., 2010). The results were in contrast to a recent finding of *increased* thickness of superior temporal cortex in Dancers and musicians (Karpati et al., 2017), as well as to prior work examining brain volume in other types of motor training, which consistently indicates increased brain volume in motor regions (e.g., Colcombe et al., 2006; Boyke et al., 2008; Jäncke et al., 2009). Specifically, when compared with handball players, professional ballet dancers had *increased* brain volume in the motor area of the foot, suggesting a sport-specific neuroadaptation (Meier et al., 2016). Therefore, it is unclear whether Hänggi et al.’s (2010) findings on brain volume in ballet Dancers were related to the Dancers’ significantly lower body mass index (BMI) compared to Non-Dancers, or to the specificity to ballet training. The results have yet to be replicated in the literature.

In addition to differences in brain volume, Hänggi et al. (2010) found that Dancers had decreased fractional anisotropy (FA) in the corpus callosum, premotor, and lateral prefrontal white matter compared to Non-Dancers. Similar findings have been established in other motor-trained groups including professional gymnasts (Huang et al., 2015). A more recent investigation examining 20 versatile-trained Dancers and 20 Non-Dancers indicated no differences in FA between groups; however, Dancers had significantly decreased mode of anisotropy in ventral prefrontal white matter, in the corticospinal tract, and in the superior longitudinal fasciculus (Giacosa et al., 2016).

Interestingly, diffusion anisotropy is a measure of white matter integrity and fiber organization, and decreases in anisotropy in adulthood have usually been associated with aging and neurological diseases (Horsfield and Jones, 2002; Burzynska et al., 2010). However, there is growing evidence that reduction in FA in young adults may not reflect white matter degeneration, but rather reflect greater axon diameter or different patterns of crossing fibers (Barazany et al., 2009; Alexander et al., 2010). For example, only two sessions of a dynamic whole-body balancing task resulted in decreases in FA in young healthy adults (Taubert et al., 2010).

### Dance Action Observation Network (AON)

Several researchers have investigated how dance proficiency affects brain function in the action observation network (AON) when watching others perform dance during a functional MRI. For instance, Calvo-Merino et al. (2005) found that brain activations within the AON were significantly greater while viewing familiar movements compared to unfamiliar movements among a small sample (*N* = 9) of expert capoeira Dancers. Specifically, the researchers found greater bilateral activations in premotor, intraparietal, posterior superior temporal and parietal cortices, as well as in medial prefrontal, cingulate, and parahippocampal gyri when experts viewed movements that they had been trained to perform compared to movements they had not been trained to perform (Calvo-Merino et al., 2005). Similarly, Cross et al. (2006) found in an fMRI study that brain activity associated with observing and imagining movements in expert Dancers was related to how much experience participants had with the dance steps, and how highly they rated their ability to perform them. These results indicate that the AON integrates observed actions of others with an individual’s personal motor repertoire, and that expertise and motor repertoire enhances brain processes of action observation in experts.

### Dance Training and Functional Connectivity (FC)

Evidence indicates that FC of the cortico-basal ganglia motor learning loops is also different in expert Dancers compared to people with no dance training. Cortico-basal ganglia loops are essential in dance training and execution as they control body posture, movement, and action selection (Nambu, 2004). Li et al. (2015) examined resting state connectivity density and FC in a sample of 28 expert modern Dancers and 33 Non-Dancers. Dancers showed increased connectivity density in cortico-basal ganglia loops that govern motor control and integration in the precentral and postcentral gyri, and in the bilateral putamen. In addition, FC was tested within regions showing differences in connectivity density. Dancers had enhanced FC between the middle cingulate cortex and the bilateral putamen, and between the precentral and postcentral gyri (Li et al., 2015). The effects of dance training on the entire motor learning network or within regions involved in dance processing remain unknown.

### Need for Synthesis

Studies on the effects of motor training, and particularly dance training on cognition as well as brain structure and function have stirred much discussion in the literature. However, the lack of cohesion and replication across studies warrants further investigation if dance is to be used therapeutically. Historically, the effects of dance training on the brain and cognition have been modular, focusing narrowly on single aspects of what is clearly a complex puzzle. Thus, it is unclear whether dance-related structural and functional changes in the brain are related, or if these neural changes are behaviorally functional, i.e., related to dance skill and cognition. Additionally, sample differences with respect to BMI or style of dance training make the synthesis of findings across studies challenging at best. Collectively, the existing literature examining the long-term effects of dance training on the healthy brain is insufficient for planning targeted interventions in clinical populations.

**FIGURE 1:**
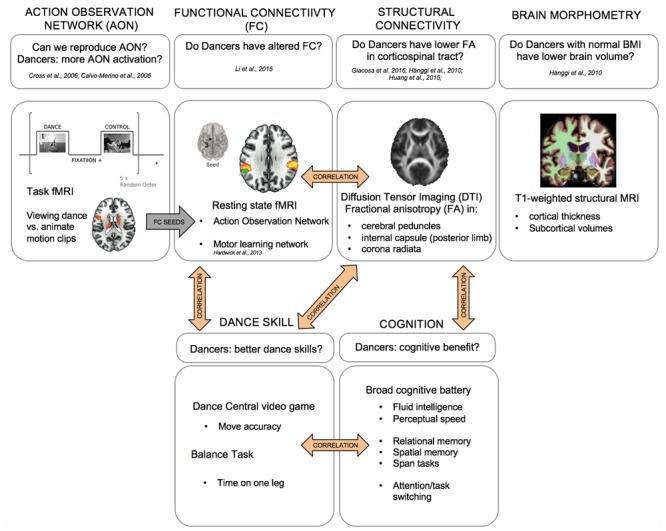
Multimodal approach to studying the structural and neural correlates of long-term expert dance training in the young human brain. **Top**: MRI imaging, **bottom**: cognitive and dance skill assessments.

The aim of the current study is to provide a comprehensive description of the effects of versatile, expert-level dance training on the structure and function of the healthy human brain, and on broadly defined cognition. To this aim, we examined a group of versatile-trained expert-level dancers as a model of long-term, high-intensity dance practice exposure. Our goal was to examine the long-term effects of dance training that may be more representative of a typical mixed-methods dance training intervention program. The unique characteristics of our study design are: multimodal neuroimaging methods, objective measures of dance skill and balance, broad cognitive assessment (well-validated tests of perceptual speed and fluid intelligence abilities, as well as tests of working memory span, relational and spatial memory, and executive function, namely task switching), and an fMRI task designed to map processing of dance stimuli. We also carefully screened our participants for healthy BMI, history of anorexia, and history of weight loss in order to control for possible effects of nutrition on brain structure and function (Hänggi et al., 2010). **Figure 1** summarizes the multimodal approach taken in this study, which (1) provides a unique replication of previous findings from multiple single-modality studies, (2) offers multimodal integration including structure-function relationships and FC analyses based on regions identified in task-based fMRI, and (3) relates multimodal data to laboratory tests of dance skill and balance, and a broad cognitive battery.

### Hypotheses

We expected Dancers to have superior performance on the Dance central video game and balance task when compared to Non-Dancers. With respect to cognitive data our aim was threefold: (1) to test whether there is a transfer from dance training to general cognitive abilities by comparing Dancers and Non-Dancers on a broad array of cognitive tests, (2) to test whether whole-body motor- and sequence-memory skills, trained by dancing, transfer to laboratory tasks of working memory span (Starkes et al., 1987, 1990), and (3) to test the hypothesis that dance-related increases in physical activity (Erickson et al., 2011) and spatial learning (Maguire et al., 2006) may enhance hippocampal function and result in improved spatial- and relational-memory.

As our Dancers and Non-Dancers were matched on BMI, we considered three alternative hypotheses with respect to cortical thickness and subcortical brain volumes: (1) Dancers would have reduced gray and white matter volume in regions similar to those indicated by Hänggi et al. (2010), supporting the notion that neuroplasticity resulting from expert dance training is related to lesser brain volume in the sensorimotor areas (James et al., 2013); (2) there would be no difference between Dancers and Non-Dancers in brain volume; and (3) Dancers would have greater brain tissue volume in motor regions or in the multimodal superior temporal regions (Karpati et al., 2017), suggesting that expert dance training follows the same pattern as other motor training routines (e.g., jugglers and golfers; Boyke et al., 2008; Jäncke et al., 2009).

With respect to white matter integrity, we predicted that Dancers would have lower FA along the corticospinal tract (cerebral peduncles, posterior limb of the internal capsule, and upper corona radiata) as well as the superior longitudinal fasciculus (Hänggi et al., 2010; Huang et al., 2015; Giacosa et al., 2016) when compared to Non-Dancers.

With respect to brain function, we hypothesized that Dancers would have greater activation of the AON and greater resting state FC of the AON and motor learning networks, including the cortico-basal ganglia loop, when viewing modern dance sequences as compared with Non-Dancers.

Finally, we expected structural and functional brain characteristics to be correlated with each other and with dance skill and balance. The approach and hypotheses are summarized in **Figure 1**.

## Materials and Methods

### Participants and Recruitment

We recruited female expert Dancers from the University of Illinois at Urbana–Champaign (UIUC) Dance Department by distributing fliers and via their dancing teachers, as well as among other UIUC dance clubs. The age-, BMI-, and education-matched Non-Dancers were subsequently recruited via fliers and student mailing lists at the University of Illinois. Only females were included in this study to minimize the effects of sex-related differences in brain size, body mass and composition, and sex hormones. We aimed to examine 20 Dancers and 20 Non-Dancers. A University of Illinois Institutional Review Board approved the study, and written informed consent was obtained from all participants and the study was performed in accordance with the 1964 Declaration of Helsinki. Participants received financial reimbursement.

After being contacted by potential participants, we sent them a pre-screening survey with basic demographic and medical history questions, as well as a survey about their dance experience and physical activity. The general inclusion criteria were: age 18–33, female, right-handed, and BMI between 18.5 and 24.9 kg/m^2^. Dancers were required to have more than 10 years of total dancing experience or >10 h/week of current training intensity for at least 3 years. This included all types of classes, including teaching dancing classes. This way we included both graduate dance students who have more years of experience but may currently dance less intensively (focus on choreography), as well as undergraduate students who have fewer years of experience but undergo very intense training. Non-Dancer participants were required to have no history of dance training or participation in martial arts, yoga, or gymnastics other than short-term (up to 3 years total) and casual participation (<2 h/week). See the Supplementary Material I for the detailed exclusion and inclusion criteria. After the written survey screening, the eligible candidate participants were contacted by phone by a trained lab assistant, who asked more detailed demographic, medical, and dance training-related questions. If the telephone interview confirmed eligibility, the participant was assigned a study number and scheduled for the Testing Session 1.

Testing Session 1 included: The Edinburgh Handedness Questionnaire, height, weight, and resting heart rate measurements, a vision test (color blindness, near/far), a mock MRI to ensure the success of the real MRI session, the fluid abilities and perceptual speed sections of the Virginia Cognitive Aging Project, and the Dance Central Video game. Testing Session 2 was scheduled within 1–2 weeks after Session 1. Testing Session 2 included an MRI session (structural MPRAGE, resting state, Dance task functional MRI, gradient field map, diffusion imaging, and MR elastography), and out-of-scanner neuropsychological testing: the face-scene relational memory task, spatial working memory, span tasks, and the balance task. Supplementary Material II summarizes the flow of the participants, explaining how we arrived at the final sample size.

One Dancer reported smoking, one had a thyroid problem and was on a relevant medication, and two reported previously being overweight (but not currently). 14/20 Dancers were enrolled full-time in the Department of Dance, and the remaining 7 were dancing within other dance clubs in the town. In the Dancer group, 15/20 were undergraduates, 4/20 were graduate students, and 1/20 reported “other” status of current education. In the Non-dancer group, 16/20 were undergraduates, 2/20 were graduate students, and 2/20 reported “other.” 4/20 Non-Dancers reported being enrolled in some casual university classes like yoga, Zumba, ballroom dance, with no more than 1 year of total experience. Ten Dancers and eight Non-Dancers were on a birth control pill. Dancers reported an average of four alcoholic drinks per week, (*SD* = 4) while Non-Dancers reported an average of 2 (*SD* = 3), and this difference was significant (*p* = 0.041). Seven Dancers and five Non-Dancers reported some prior Kinect video game experience.

### Cognitive Assessment and Analysis

We administered a part of a standardized cognitive battery as described in the Virginia Cognitive Aging Project (Salthouse and Ferrer-Caja, 2003; Salthouse, 2004, 2005, 2010) to measure latent constructs of fluid intelligence (matrix reasoning, paper folding, spatial relations, letter sets, form boards, Shipley abstraction) and perceptual speed (pattern and letter comparison, digit symbol substitution; Supplementary Material III). We did not include the vocabulary and memory parts of the battery due to time constraints. Additionally, (1) we did not exclude non-native English speakers as the dance community tends to be international, which could potentially bias the vocabulary component, (2) there is no evidence to state a hypothesis about the crystallized knowledge differences between Dancers and Non-Dancers, and (3) we replaced the memory tasks with tests of relational-, sequential-, and spatial-memory that depend less on English fluency and better probe spatial- or relational-memory associated with the hippocampus, which is also involved in spatial memory. The trail making test was used to assess task switching and memory (Supplementary Material III; Reitan, 1992). Computer-based tasks were programmed in E-prime version 1.1 (Psychology Software Tools, Pittsburgh, PA, United States) and administered on computers with 17″ cathode ray tube monitors.

To obtain components representing the two cognitive constructs (fluid intelligence and speed), we performed principal component analysis (PCA) with varimax rotation on the nine tasks. The resulting constructs are presented in Supplementary Material III and the component scores were saved as variables.

### Dance Central Video Game

In addition to the survey about Dancers’ training history and intensity, we assessed the dance mimicry ability of all participants using the commercial video game Dance Central 2 (Harmonix Music Systems 2011) for the XBox 360 Kinect^TM^ console. Galna et al. (2014) showed that results from Kinect are strongly related to these obtained with the gold-standard 3D Vicon system, especially for gross movements, such as whole-limb and torso movements. Others recently reported good-to-excellent accuracy for the Kinect system with respect to the Vicon for clinical parameters (Pfister et al., 2014; Otte et al., 2016), concluding that the Kinect has the potential to be used as a reliable and valid clinical measurement tool. Despite possible differences in motion detection accuracy with the Kinect from more sophisticated setups, we argue that this measure was suitable for general assessment of dance ability. Moreover, we used the same measures for Dancers and Non-Dancers, and any motion detection accuracy issues would affect the two groups to the same extent.

Participants were instructed to mirror dancing moves displayed on a screen as accurately as possible, and their performance and movement accuracy were automatically scored by the motion detector. To ensure consistency, all participants played the same song “Dip It Low” by Christina Milian (song level difficulty: medium, mode: hard). Each participant danced this song six consecutive times to correct for possible bias in a single motion detector trial. The percent of correct moves was recorded after each trial. The overall dance game testing lasted up to 20 min. We assessed the total performance as the PCA score from all six trials.

### Balance Task

The task measured balance ability and motor coordination skill. We carried out a modified (more difficult) version of one of tasks within a battery testing risk of falls in older populations (Lord et al., 2003). Adding difficulty was necessary as we observed ceiling effects in healthy younger adults in both normal standing and one-leg standing conditions. A 50 cm × 60 cm × 15 cm foam pad was placed on flat smooth surface in the laboratory, with at least 1.5 m of free space around the pad on each side. Two testers assisted in this task to ensure participant safety, while the third measured time with a timer. The participant was asked to step in the middle of the foam and to stand for a few seconds on each leg, with eyes open, to adjust to the foam pad and decide on which leg she felt more stable. The participant was asked to stand on her dominant leg (with the other one lifted in the air), close her eyes, and stand as long as possible. There were three trials and the longest (best) score was recorded as the participant’s final score. If the participant exceeded 1min, the trial was terminated and a score of 60 given.

### MRI Acquisition

All MRI images were acquired in a single session on a 3T Siemens Tim Trio system with 45 mT/m gradients and 200 T/m/sec slew rates (Siemens, Erlangen, Germany). Structural MR scans were acquired using a 3D MPRAGE T1-weighted sequence (TR = 1900 ms; TE = 2.32 ms; TI: 900 ms; flip angle = 9°; matrix = 256 × 256; FOV = 230 mm; 192 slices; resolution = 0.9 mm × 0.9 mm × 0.9 mm; GRAPPA acceleration factor 2). DTI images were acquired with a twice-refocused spin echo single-shot Echo Planar Imaging sequence. The protocol consisted of a set of 30 non-collinear diffusion-weighted acquisitions with *b*-value = 1000s/mm^2^ and two T2-weighted *b*-value = 0 s/mm^2^ acquisitions, repeated two times (TR/TE = 10000/98 ms, 128 × 128 matrix, 1.9 mm × 1.9 mm in-plane resolution, GRAPPA acceleration factor 2, and bandwidth of 1698 Hz/Px, comprising 72 2-mm-thick slices for full brain coverage), acquired with no rotation and no interslice gap. Whole-brain functional MRI data were collected using a standard EPI sequence (TR/TE = 2000/25 ms, flip angle 90°, voxel size 2.5 mm × 2.5 mm × 3.0 mm, interslice gap 0.15, 38 slices acquired in ascending order approximately axial to the bicommissural plane). Three dummy volumes preceded the resting state acquisition to achieve a steady state of tissue magnetization. Resting state lasted 8 min 6 s (240 volumes saved for analysis) and each run of the task fMRI lasted approximately 5 min. Gradient Field Map images were acquired using a standard EPI sequence (TR/TE1/TE2 = 500 ms/5 ms/7.46 ms, flip angle 30°, voxel size 2.5 mm × 2.5 mm × 3.0 mm, interslice gap 0.15, 38 slices acquired in ascending order approximately axial to the bicommissural plane).

### Cortical Thickness and Subcortical Volume Analysis (Freesurfer)

Automated brain tissue segmentation and reconstruction of cortical models were performed on T1-weighted images using Freesurfer software, version 5.3^1^. Surface-based methods have been shown to be superior in reflecting the surface organization of the cortex and subcortical segmentation when compared to gray-matter density or gyral/lobar volume (Hutton et al., 2009). In brief, individual T1-weighted images underwent non-brain tissue removal, Talairach transformation, creation of representations of the gray/white matter boundaries (Dale and Sereno, 1993; Fischl et al., 1999a,b) and calculation of the cortical thickness as the distance between the gray/white matter boundary and the pial surface at each point across the cortical mantle (Fischl and Dale, 2000). We carefully screened all surface reconstructions to evaluate the success and plausibility of the automatically processed results, as recommended by the software developers. Next, cortical thickness maps were inflated (Fischl et al., 1999a,b), smoothed using a circularly symmetric Gaussian kernel across the surface with a full width at half maximum of 10 mm, and registered to a spherical atlas to match individual cortical folding patterns across subjects (Fischl et al., 1999a,b).

We compared cortical thickness between Dancers and Non-Dancers with a two-sample *t*-test at each vertex (measurement point at the cortical mantle). All results are shown on inflated standard cortical surface maps with a *p*-value < 0.01, uncorrected (see Supplementary Material IV). The volume of subcortical structures and white matter was extracted separately and compared between Dancers and Non-Dancers with an independent samples *t*-test in SPSS, uncorrected (v.23, SPSS Inc., Chicago, IL, United States).

### Diffusion Imaging Analysis

Diffusion imaging allows inferences about white matter microstructure *in vivo* by quantifying the magnitude and directionality of the diffusion of water within a tissue. FA, a measure of the directional dependence of diffusion, reflects the level of white matter integrity within a voxel (Beaulieu, 2002). Diffusion data were processed and FA maps were computed using the FSL Diffusion Toolbox (FDT) v.3.0 in a standard multistep procedure with default settings. Images were carefully inspected at each level of preprocessing, including visual inspection of each raw diffusion data volume by AZB and lab members mentioned in the acknowledgments.

We used Tract-Based Spatial Statistics (Smith et al., 2007) within FSL v5.0.1 with default settings, to create a representation of main white matter tracts common to all subjects. Next, we averaged the FA values from all the main white matter tracts for each participant to obtain global FA. In addition, we extracted FA values from four regions representing the superior corona radiata, posterior limb of the internal capsule, cerebral peduncles, and superior longitudinal fasciculus, as described in Burzynska et al. (2013).

In addition, an exploratory analysis comparing FA across the whole white matter skeleton between Dancers and Non-Dancers was carried out in randomize, a voxel-wise permutation-based (5000 permutations) inference (Nichols and Holmes, 2002) with the threshold-free cluster enhancement option (corrected).

### fMRI Task: Mapping the AON Related to Dance

The fMRI task was designed to elicit brain activity patterns reflecting dance-based sensorimotor representation by inducing dance imagery while watching modern dance videoclips, similar to Di Nota et al. (2016). To identify brain activity related to dance stimuli, but not to animate motion and object recognition, the task design contrasted modern dance clips with emotionally neutral videos of animate motion that were of similar pace and complexity to the dance videos. During the task, participants watched color clips of either modern dance solos (Dance condition) or animate motion (Control condition). The experiment consisted of three runs with different 16–20 s clips from the same five dance or animal videos separated by a 10–14 s fixation period in a block design. As a result, the longest possible run lasted 322 s and the shortest lasted 268 s. The order and duration of the video clips and the duration of each fixation were randomized to maximize the experimental power and design efficiency (Huettel et al., 2004). The fMRI task was created in E-Prime 2.0.

While they watched dance videos, all participants were instructed to imagine performing the dance sequence. Dance performance videos were selected based on the following criteria: (1) They were solo performances in a Modern/Contemporary style by a female dancer; (2) The dancer’s entire body was visible at all times throughout the video; and (3) The level of difficulty in terms of choreography was such that the moves could theoretically be performed by both Dancers and Non-Dancers (i.e., not too hard). Therefore, the instruction was equally feasible for both groups, although for Dancers this instruction resembled remembering the choreographed sequence during dance training; (4) The videos displayed a moderately bright color scheme with approximately the same colors as the other selected videos; and (5) Each of the five selected dance videos was split into three clips for each of the three runs. Each run included a different section of each dance video.

**FIGURE 2:**
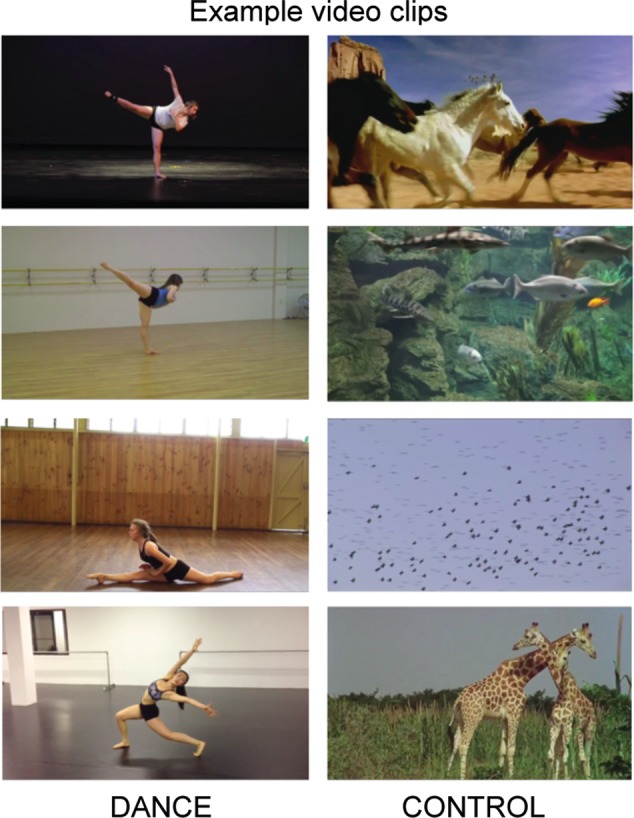
Examples of Dance and Control videoclips used in fMRI task for mapping the action observation network (AON) of dance stimuli.

Similarly, the animate control videos were selected based on the following criteria: (1) They contained only animals (no humans); (2) The animals were moving throughout the video at similar speed as in dance videos; (3) The videos contained neutral stimuli that were not emotionally charged; (4) The videos displayed a moderately bright color scheme with approximately the same colors as the other selected videos; and (5) Each of the five selected animate motion videos was split into three clips for each of the three runs. Each run included a different section of each animate motion video. The role of the Control condition was to elicit brain activity related to animate motion, object recognition and general visual perception. Subtracting animate motion brain activity from the dance condition should yield a specific “action observation network” related to dance information processing. Examples of material from Dance and Control video clips are presented in **Figure 2**.

We performed thorough quality checks on the raw data including estimation of motion displacement along three axes from MCFLIRT. Runs with motion above 3 mm (the largest dimension of the voxel size) that affected several volumes were excluded. Two participants, both Dancers, had significant motion in one of the three runs. In these cases, the run with excessive motion was excluded, and the other two runs were each entered into analyses twice and averaged together to maintain power. FSLMotionOutliers was used to account for non-linear artifacts and slice-by-slice movements induced by large, rapid motions, as well as changes in intensity between volumes (Jenkinson et al., 2012). The motion outliers identified using the output of this program were included as covariates in the first level analysis (below). We also accounted for intensity spikes, calculated as any difference greater than 1.5% between the mean intensity of all images and the mean intensity of the specific volume.

Registration of functional images to high-resolution T1-weighted images was performed using Boundary-Based Registration (Greve and Fischl, 2009) with normal search. Next, T1-weighted images were registered to standard space (MNI 152_T1 2 mm × 2 mm × 2 mm) in a two-step process. First, we used linear registration (affine, normal search, 12 degrees of freedom) in FLIRT (Jenkinson and Smith, 2001; Jenkinson et al., 2002), which was further refined with non-linear registration (FNIRT; Andersson et al., 2007) with a warp resolution of 8 mm. The registration matrices were then combined to transform the functional data of each run to the MNI 152_T1 template.

The images were unwarped (corrected for distortion caused by local magnetic susceptibility differences) using the gradient field map data (FUGUE in FSL version 5.0.0). Then, hemodynamic response function data underwent convolution using a double gamma function accounting for the motion parameters as described above. Data from each subject’s runs were combined using second level analysis in FEAT in FSL version 5.0.0 (Jenkinson et al., 2012) by using fixed model, group average GLM analysis. Finally, group analysis was performed using a higher-level mixed effects model, FLAME (FMRIB’s Local Analysis of Mixed Effects), which forced the random effects variance to zero (Smith et al., 2004; Woolrich et al., 2004). The contrast of interest was Dance > Control condition for Dancers > Non-Dancers. The other contrasts (e.g., Dance > fixation, Control > fixation, Control > Dance) are presented in Supplementary Material VI. Significant regions were identified as clusters determined by *z* > 2.3 (Z; Gaussian T/F) and a cluster significance threshold of *p* = 0.05 (corrected; Worsley, 2003). To restrict the final group analyses to the gray matter, we masked our functional data with the GM tissue prior provided in FSL (threshold at probability > 0.20).

### FC Analysis of the Resting State MRI

Resting state functional images were preprocessed using the SPM12 toolbox (Wellcome Department of Imaging Neuroscience, Institute of Neurology, London, United Kingdom) and CONN Functional Connectivity Toolbox v. 15.f^2^ (Whitfield-Gabrieli and Nieto-Castanon, 2012) running on Matlab 8.3 (R2014a) (Mathworks, Natick, MA, United States). Note that co-authors in this collaborative multimodal project were using the software of their choice to preprocess the data. Given that outcomes of fMRI analyses depend more on preprocessing steps than on a particular software package (e.g., Carp, 2012), we describe each preprocessing step to allow replication of our findings.

The first five functional volumes were removed to allow for signal equilibration. We performed slice-timing correction to correct for acquisition time and spatial realignment to the mean functional image using six rigid-body parameters (three translations: X, Y, Z; three rotations: pitch, roll, yaw) to correct for head motions. Additionally, outlier volumes were identified using the artifact detection (ART) toolbox ^3^. Volumes were tagged as outliers when a frame-wise displacement was above 0.5 mm or if the global mean signal intensity was above 3 SD from the mean intensity of the functional image. Three participants (all Dancers) were excluded from further analyses at this point due to excessive head motion, defined as >10% of volume staged as outliers.

Next, non-neural signal (physiological noise) was estimated from regions in white matter and cerebrospinal fluid using anatomical component-based correction (aCompCor) implemented in CONN (Behzadi et al., 2007; Whitfield-Gabrieli and Nieto-Castanon, 2012). The principal components of the signal from white matter (three PCA parameters), CSF (three PCA parameters), as well as motion parameters (six rigid-body parameters), outlier volumes identified with ART, and a linear detrending term were regressed out as covariates in a linear regression model for each participant. The 0.008–0.09 Hz band-pass filter was applied in order to remove the effect of high-frequency noise and low-frequency drift (Lowe et al., 1998).

#### FC Analysis of the AON

We used the conjunction contrast of both Dancers and Non-Dancers as the representation of the AON (as in Calvo-Merino et al., 2005; Cross et al., 2006). Within the map of significant (*z* > 2.3) activity (Dance > Control condition) we created 4 mm radius spherical seeds around four local maxima, and the other four seeds were added to ensure the symmetry of the analyses (full list of seed coordinates: Supplementary Material VII). In addition, we carried out analogous analysis within the regions that showed greater BOLD signal increase in Dance > Control condition in Dancers compared to Non-Dancers (full list of seed coordinates: Supplementary Material VII). Residual BOLD time-courses were extracted from the seed regions and Pearson’s correlation coefficients were calculated between each seed time-course and the time-courses of the remaining voxels in the brain. Fisher’s transformation was applied to convert correlation coefficients to normally distributed *z*-scores to allow for second-level group comparisons. We calculated an independent samples *t*-test for each seed to assess differences in seed-voxel connectivity between Dancers and Non-Dancers. Cluster-wise significance threshold (two-tailed) *P*_FDR_ < 0.05 was retained for all analyses. A Bonferroni correction was applied by adjusting the *p*-value: 0.05/number of tests performed. We carried out seed-to-seed, seed-to-mask, and seed-to-entire-brain tests in both analyses.

#### FC of the Motor Learning Network

Ten 4-mm radius spherical seeds were created using the MarsBaR toolbox^4^ on the basis of coordinates from a recent motor learning meta-analysis (Hardwick et al., 2013). These areas showed consistent activation across various motor learning tasks including both sensorimotor tasks and serial response time task (Hardwick et al., 2013) and included regions localized to bilateral primary motor and supplementary cortex, putamen, left superior parietal lobule, left thalamus, left somatosensory cortex, and left dorsal premotor cortex (seed center coordinates are listed in Supplementary Material VII). We did not analyze the seeds located in cerebellum due to variable coverage of cerebellum in our EPI Images.

## Results

### Experience and Skill

Dancers reported a significantly greater number of hours spent on dancing practice per week, and years of regular/intensive practice compared to Non-Dancers (**Table 1**). Note that few of the Dancers reported shorter periods of intense training (minimum was 3 years), but these were the individuals who were undergoing the most intense current training. Therefore, our sample represented expert dancers with some variability in training patterns. Years of practice for Non-Dancers reported in **Table 1** refer to casual-, not expert-level training.

**Table 1:** Demographics, dance experience, and neurocognitive performance.

	Dancers			Non-Dancers			
	*n*	Mean	*SD*	*n*	Mean	*SD*	*p*-value
Age	20	21.6	4.2	20	21.3	2.4	0.051
BMI [kg/m^2^]	20	22.3	1.8	20	22.0	2.1	0.603
Education	20	15.15	2.8	20	15.35	1.7	0.081
Resting HR	19	70	9	20	72	13	0.598
Dance experience and ability							
Age started dancing	20	6	4	n.a.	n.a.	n.a.	n.a.
		Range	2.5–18				
Years of practice	20	12	6	19	0.4	0.76	<0.001
		Range	3–20	Range	0–3		
Hours/week	20	14	8	19	0.5	1.4	<0.001
		Range	1.5–30	Range	0–6		
Balance							
Best trial (sec)	19	19	13	19	10	6	0.012
Dance Game							
%PCA^∗∗^	20	0.52	0.93	20	–0.54	0.82	0.001
Neuropsychological tests							
Fluid PCA^∗^	20	0.084	0.72	20	–0.031	1.23	0.702
Speed PCA^∗^	20	–0.24	0.87	20	0.23	1.08	0.132
Trail making							
A	20	25	8	20	24	8	0.741
B	20	49	10	20	51	14	0.465
B–A	20	24	9	20	28	11	0.263
SPWM ACC							
1-back	19	0.85	0.12	18	0.89	0.09	0.389
2-back	19	0.92	0.06	18	0.93	0.04	0.700
3-back	19	0.88	0.06	18	0.85	0.11	0.405
4-back	19	0.86	0.10	18	0.87	0.06	0.585
SPWM RT							
1-back	19	874	199	18	743	134	0.025^+^
2-back	19	764	138	18	676	94	0.033^+^
3-back	19	807	130	18	703	102	0.011^+^
4-back	19	851	168	18	735	84	0.013^+^
ACC PCA	19	–0.05	1.0	18	0.05	0.90	0.765
RT PCA	19	0.37	1.1	18	–0.37	0.67	0.012^+^
Span							
Forward	19	5.2	1.1	19	5.1	1.1	0.885
Backward	19	4.6	1.3	19	4.6	1.3	1.00
Relational memory							
Face-scene task (d’)	19	0.23	0.12	18	0.31	0.12	0.044^+^
DTI							
PLIC	18	0.6903	0.0208	19	0.7041	0.018	0.039^+^
SCR	18	0.6187	0.0153	19	0.6182	0.024	0.942
CP	18	0.7774	0.0169	19	0.7803	0.025	0.682
SLF	18	0.5104	0.0203	19	0.520	0.017	0.125

*^∗^PCA: Principal Component Analysis; On a task level, the Dancers completed less items in the Digit Symbol (87 vs. 98 in Non-Dancers, *p* = 0.020), and Dancers had higher accuracy on letter sets (13 vs. 12 in Non-Dancers, *p* = 0.030). ^∗∗^Calculated from all six trials. ACC, accuracy; RT, reaction time; PLIC, posterior limb of internal capsule; SCR, superior corona radiata; CP, cerebral peduncles; SLF, superior longitudinal fasciculus. In Bold: significance *p* < 0.05, uncorrected. **^+^**Not significant at Bonferroni-corrected *p* = 0.05/4 for DTI or *p* = 0.05/9 for cognitive tasks.*

Next, we used objective measures of dance ability (Dance Central video game) and balance to confirm group identity and obtain a continuous measure of dance skills for the entire sample. Dancers’ overall percent of correct moves and time spent balancing on one leg was greater than Non-Dancers’ (**Table 1**).

Finally, we investigated whether the length or intensity of dance training was related to performance on the two laboratory tests of motor skill. It is important to know how the laboratory tasks map onto training length and intensity before studying their relationship with brain measures. First, by correlating age with several variables of interest, we found that: (1) older Dancers tended to have less intense current practice, possibly due to being graduate students and doing more teaching/choreography (*r* = -0.494, *p* = 0.027); (2) older Dancers also performed worse on Dance Central (*r* = -0.63, *p* = 0.003); and (3) older Dancers tended to have less video game experience (*r* = -0.40, *p* = 0.100). Although current dancing intensity was strongly associated with Dance Central performance (*r* = 0.48, *p* = 0.034), this relationship remained at trend level after controlling for gaming experience or age, most likely due to the aforementioned relations between age, current practice, and video game experience in Dancers. After controlling for age, there was a significant relationship between years of regular practice and Dance Central performance (*r* = 0.55, *p* = 0.017). Balance was not significantly related to self-reported dance experience measures in Dancers.

Within the control group, we found that years of casual practice was positively associated with Dance Central performance (*r* = 0.43, *p* = 0.058), and this remained a trend after controlling for Kinect game experience or age. In addition, the number of current practice hours per week was positively associated with performance on the balance task (*r* = 0.55, *p* = 0.016). Importantly, there was no group difference in gaming experience between Dancers and Non-Dancers.

Together, we confirmed that the Dancers indeed had years-long practice in dance, which was related to their superior performance on dance-related laboratory tasks. Years of experience in motor activities was linked to greater Dance Central scores in both groups.

### Cognitive Performance

We compared Dancers and Non-Dancers on a broad battery of cognitive tasks to check whether dance training may be associated with transfer to different cognitive domains, with a focus on spatial, sequential, and relational memory. We observed no group differences in most cognitive domains, including fluid intelligence, processing speed, spatial working memory accuracy, working memory span, and task switching. Interestingly, Dancers were significantly slower on the spatial working memory task and had a lower *d*′ (the difference of the *z*-scores of the hits and false alarms) on the relational memory face-scene task (**Table 1**). However, these differences were not significant following Bonferroni correction for multiple comparisons given the number of cognitive tasks.

We investigated the relationships among cognitive outcomes showing group differences (*d*′, spatial working memory latency), dance skill measures, and FA in the posterior limb of the internal capsule (see White Matter Microstructure for details). We found a negative relationship between *d*′ and video game performance in the whole sample (*r* = -0.42, *p* = 0.011, *n* = 37), and a weak positive relationship between *d*′ and PLIC FA (*r* = 0.33, *p* = 0.048, *n* = 36). In other words, better Dance Central performance was related to lower performance of relational memory, and lower relational memory was linked to lower integrity in the PLIC. We found no relationships for the entire group between PLIC FA and spatial working memory latency.

### Brain Volume

We compared Dancers and Non-Dancers with respect to their cortical thickness and subcortical volumes. Total intracranial volume did not differ between groups (*p* = 0.138). Surface-based analysis showed only weak (*p* < 0.01, uncorrected) differences between groups: Dancers showed a trend toward a thinner cortex in the right precentral, postcentral, superior temporal, and posterior cingulate gyri (Supplementary Material IV). There were no significant differences in subcortical gray and white matter volumes between the groups, even after controlling for age.

### White Matter Microstructure

We compared white matter microstructure between Dancers and Non-Dancers in the four regions representing core sections of the corticospinal tract and the fronto-parietal connection. We found a significant difference in FA in only one region: the PLIC, where Dancers had lower FA than Non-Dancers (**Table 1**). The exploratory whole-white matter analysis in TBSS confirmed that there was a trend toward lower FA in Dancers in the left corticospinal tract (Supplementary Material V). Interestingly, balance and Dance Central performance were not related to PLIC FA in either of the groups or in whole sample combined, nor to hours of weekly practice or years of experience in Dancers.

### Activation of the AON during Dance Observation

**FIGURE 3:**
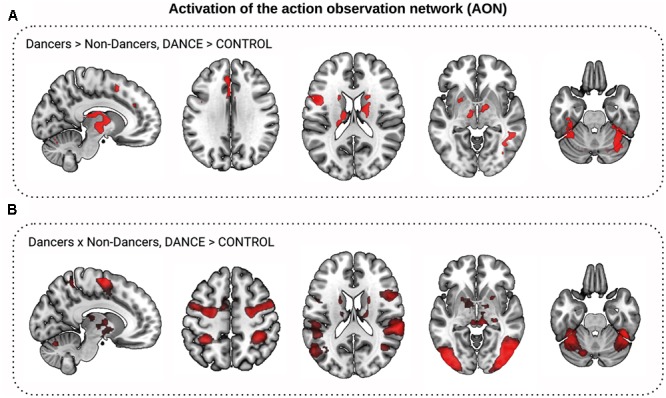
Activation of the action observation network (AON) during dance observation fMRI task. **(A,B)** Show comparison of Dance to Control video clips observation conditions (DANCE > CONTROL). **(A)** Dancers activated the AON to a greater extent than Non-Dancers: Dancers showed greater increase in the BOLD signal to Dance than to Control video clips than Non-Dancers in the basal ganglia, higher visual, precentral, and frontal cortex (in red, *z* > 2.3, corrected). **(B)** The AON for both Dancers and Non-Dancers (conjunction, in red).

We tested the hypothesis that Dancers show stronger activation of the AON than Non-Dancers. Indeed, Dancers showed a greater increase in BOLD signal for the condition Dance > Control in the left inferior frontal gyrus, premotor cortex, and putamen, in the bilateral fusiform cortex and thalamus, and in the anterior cingulate and superior frontal gyri (**Figure 3A**). There were no regions where Non-Dancers activated the AON more than Dancers. Next, we mapped the general AON by comparing the change in the BOLD signal between viewing Dance and Control videos for the full sample. Participants showed greater BOLD signal increase to Dance clips compared to Control clips in the bilateral precentral gyrus (motor and premotor cortices), supplementary motor areas, inferior frontal gyrus, superior parietal lobule, supramarginal gyrus, inferior parietal cortex, fusiform cortex, lateral occipital cortex, and the basal ganglia: putamen, thalamus, caudate, and pallidum (**Figure 3B**). Supplementary Material VI contains the results for the other contrasts.

### FC of the AON Activated by Dance Stimuli

**FIGURE 4:**
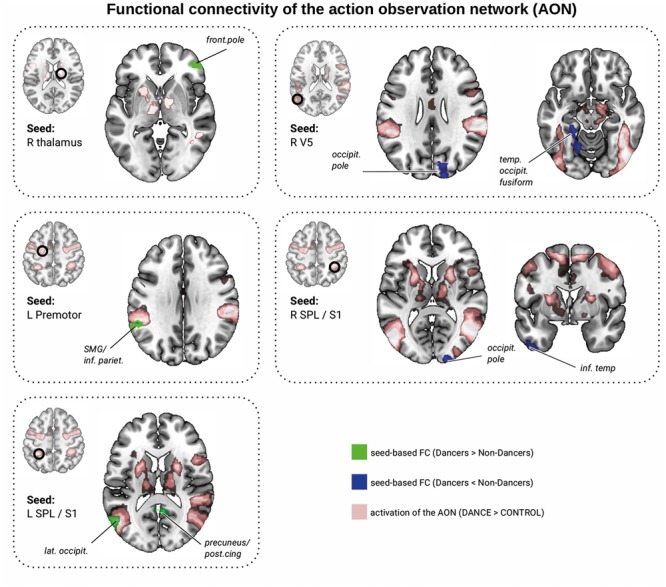
Resting-state functional connectivity differences between Dancers and Non-Dancers for seeds belonging to the action observation network (AON), representing local maxima from brain activation analysis shown in **Figure 3**. Seed ROIs are presented in the brain space and are marked with a black circle and refer to **Table 2**. Significant clusters are in green if FC Dancers > Non-Dancers, or in blue if FC Non-Dancers > Dancers. All images are overlaid on a standard 2 mm^3^ MNI brain. For full list of analyzed seed regions see the Supplementary Material VII.

We investigated group differences in functional organization related to the AON. First, we focused on regions where Dancers activated the AON to larger extent than Non-Dancers (Dance > Control, Dancers > Non-Dancers, at *z* > 2.3). There were no group differences in FC within this network (seed-to-seed analyses and seed-to-mask). In the seed-to-whole brain analysis, Dancers had greater connectivity than Non-Dancers between the right thalamus and the right frontal pole (**Figure 4** and **Table 2**, corrected). We found that individual FC scores were significantly correlated with Dance Central performance in the entire sample (**Table 2** and Supplementary Material VII and IX). In addition, there was only a trend for a negative relationship between PLIC FA and FC (*r* = -0.27, *p* = 0.113, *n* = 36).

**Table 2:** Group differences of the seed-based FC differences between Dancers and Non-Dancers (corrected).

			MNI coordinates							
Seed	Correlated area	Size (voxels)	*x*	*y*	*z*	Peak *t*-score	Cluster *p*-FDR	D vs. n-D	Dance game	Balance
**Action observation network (Dancers > Non-Dancer) (see **Figure 4**)**				
(1) R thalamus	R frontal pole	154	48	44	2	5.15	0.0163	D > nD	*r* = 0.35, *p* = 0.037, *n* = 36	–
**Action observation network (Dancers and Non-Dancer) (see **Figure 4**)**				
(2) L V5	L temporal occipital fusiform	299	–24	–34	–4	–6.10	0.0013^∗^	nD > D	*r* = -0.34, *p* = 0.42, *n* = 36	–
	R occipital pole	251	18	–88	26	–4.89	0.0020^∗^	nD > D	*r* = -0.40, *p* = 0.015, *n* = 36	*r* = -0.42, *p* = 0.037, *n* = 36
(3) L premotor	L supramarginal/inferior Pariet.	132	–54	–46	28	4.75	0.0402	D > nD	*r* = 0.32, *p* = 0.52, *n* = 35	–
(4) R SPL /S1	R occipital pole	156	20	–98	22	–5.78	0.0245	nD > D	*r* = -0.39, *p* = 0.019, *n* = 35	–
	L inferior temporal	146	–46	4	–46	–5.69	0.0245	nD > D	*r* = -0.48, *p* = 0.003, *n* = 35	–
(5) L SPL /S1	L lateral occipital	251	–56	–72	10	–5.27	0.0022	nD > D	*r* = -0.32, *p* = 0.058, *n* = 35	–
	Precuneus/posterior cingulate	132	4	–50	18	–4.50	0.0295	nD > D	–	–
**Motor learning network (after** Hardwick et al., 2013) (see **Figure 5**)				
(6) L SPL	L precentral/L postcentral	217	–48	–14	14	6.44	0.000083^∗^	D > nD	–	–
	R precentral	118	52	–2	12	6.82	0.0039^∗^	D > nD	*r* = 0.33, *p* = 0.055, *n* = 35	–
(7) R M1	R fusiform	107	34	–78	–16	6.04	0.0057	D > nD	–	*r* = 0.31, *p* = 0.067, *n* = 35
(8) R putamen	R precuneus	58	6	–46	32	4.97	0.0315	D > nD	–	*r* = 0.303, *p* = 0.076, *n* = 35
	R inferior frontal	57	56	18	18	–5.55	0.0315	nD > D	*r* = -0.49, *p* = 0.003, *n* = 35	*r* = -0.31, *p* = 0.066, *n* = 35

**FIGURE 5:**
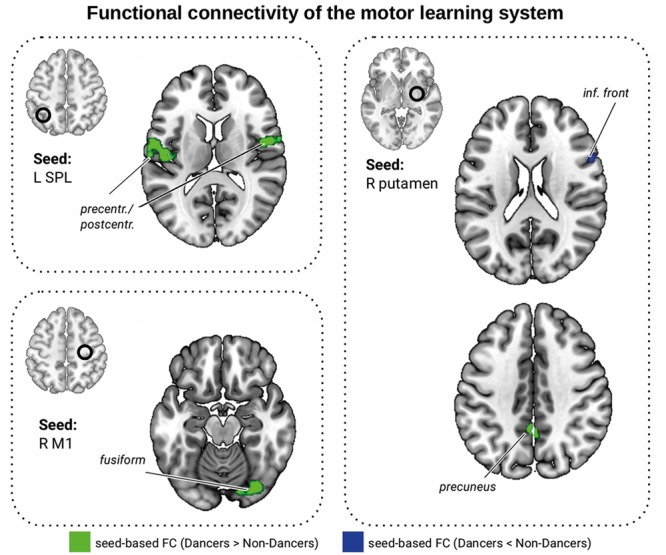
Functional connectivity differences within the motor learning network (Hardwick et al., 2013). Seed ROIs are presented in the brain space and are marked with a black circle. Green: FC Dancers > Non-Dancers, blue: FC Non-Dancers > Dancers. All images are overlaid on a standard 2 mm^3^ MNI brain. For full list of analyzed seed regions see **Table 2** and the Supplementary Material VII.

*All analyses 2-tailed, *p* < 0.05, FDR-corrected. SPL, superior parietal lobule; S1, primary somatosensory cortex; M1, primary motor cortex; D, Dancers; nD, Non-Dancers. **^∗^**Significant after Bonferroni correction at *p* < 0.05/number of tests. 1–8: seed regions, same as shown in **Figures 4, 5**.*

Next, we investigated functional organization within the AON common to all participants (Dance > Control, Dancers AND Non-Dancers, at *z* > 2.3; **Figure 4**). Again, we found no group differences in FC within this network (seed-to-seed analyses, and seed-to-mask). A seed-to-whole brain analysis revealed that Dancers had enhanced FC between left premotor cortex and left supramarginal gyrus/inferior parietal lobule, but attenuated FC in several other regions (**Figure 4** and **Table 2**, corrected). PLIC FA was not related to any of the individual FC values. However, FC from left V5 to the right occipital pole was negatively related to balance performance (**Table 2**). Dance Central performance was negatively related to most regions where Dancers had lower FC than Non-Dancers, including connections from the left V5 to temporal/occipital fusiform and left occipital pole, right SPL to occipital pole and left inferior temporal gyrus (see Supplementary Material IX for scatterplots). There was a positive trend for left premotor cortex to left supramarginal gyrus (SMG; **Table 2**), where Dancers had greater FC than Non-Dancers. Together, FC in certain regions connecting the AON to other brain regions was correlated with Dance Central performance across all participants. The direction of these relationships was consistent: in regions where there was lower FC in Dancers, the relationship with performance was also negative.

### FC of the Motor Learning Network

We compared the Dancers and Non-Dancers with respect to their FC within the motor learning network (Hardwick et al., 2013; all regions listed in Supplementary Material VII). There were no significant group differences in the seed-to-seed analysis. In the seed-to-whole brain analysis, Dancers had greater FC than Non-Dancers between the left SPL and bilateral precentral and postcentral cortex, between right primary motor cortex (M1) and right fusiform cortex, and between right putamen and precuneus, but lower FC between right putamen and right inferior frontal gyrus (IFG; **Table 2** and **Figure 5**, corrected). In additional analyses, we showed that these results were not driven by differences in head motion (Supplementary Material VIII).

We found several correlations between FC of the motor learning network and dance skill. Lower FC between right putamen and right IFG was associated with better Dance Central performance (**Table 2**; see Supplementary Material IX for selected scatterplots). There was a trend for an association between Dance Central performance and FC between left SPL and right precentral gyrus (PCG, **Table 2**). There were only trends relating balance to FC (**Table 2**). Interestingly, lower PLIC FA was associated with greater FC between left SPL and left PCG (*r* = -0.39, *p* = 0.021, *n* = 35), and with lower FC between right putamen and right IFG (*r* = 0.43, *p* = 0.010, *n* = 35). Together, the direction of these relationships was consistent: in regions where Dancers had greater FC, the relationship with dance performance was negative, and with PLIC FA was positive.

## Discussion

By comparing a group of versatile-trained, expert Dancers with age-, BMI-, and education-matched Non-Dancers, we challenged the existing findings on the effects of dance on brain structure and function. First, we showed that dance training was not related to better cognitive abilities. Second, our structural analyses showed only a trend toward thinner cortex in Dancers compared to Non-Dancers. Interestingly, we replicated the findings on lower FA in the corticospinal tract in Dancers. More importantly, we extended the evidence for the effects of dancing on functional brain organization by showing that the AON is activated to a larger extent in Dancers than in Non-Dancers. Furthermore, we showed differences in spontaneous FC of the AON as well as the motor learning network. Most of the group differences in FC between Dancers and Non-Dancers were functional: they were related to dance performance or corticospinal white matter microstructure. We discuss these neuroimaging findings with respect to prior research on adult brain plasticity and implications of for future interventions and dance therapy.

### Dance Training Enhances Motor Coordination and Balance

Our study was the first to incorporate a broad battery of cognitive tests as well as laboratory assessments of dance skill and balance, in addition to self-report measures of dance training length and intensity. This way, we were able to test the relationships between laboratory tasks and self-reported dance experience. We demonstrated that both self-reported measures and laboratory tasks clearly separated Dancers from Non-Dancers, confirming the validity of the criteria we chose for recruiting and identifying Dancers and Non-Dancers. When correlating the laboratory and self-report measures, we observed a dissociation between Dancers and Non-Dancers.

In Dancers, balance performance was not related to dance experience. In Non-Dancers, current engagement in activities requiring coordination was related to balance performance. Our findings suggest that the Dancers might have reached the ceiling for their balance performance, as neither length nor current intensity of training was related to their performance. In contrast, control participants’ performance on the balance task seemed to benefit from current casual dance practice (both length and duration). This may have significant implications for dance interventions in low-active or untrained populations, such as older adults or clinical populations. Namely, even casual participation in activities involving coordination may positively impact balance and potentially lead to reduced risk of falls (Cadore et al., 2013).

Dance Central performance was related to years of regular practice in Dancers, and to years of casual dance in Non-Dancers. Both relationships were moderated by age and gaming experience. The data suggest that exposure to dance-related activities, either at the expert- or casual-level, relates to better Dance Central game performance, which requires fast and accurate whole-body mimicking, coordination, and motor execution. The findings suggest that accumulation of motor skills at both casual- and expert-levels may lead to greater “motor reserve” that could be protective against aging and other progressive neurological disorders (Fleischman et al., 2015). This should serve as encouragement to engage in activities that build motor skills throughout the lifespan.

### Dance Has Limited Transfer to Other Cognitive Abilities

As predicted, Dancers did not differ from Non-Dancers on fluid intelligence, processing speed, spatial working memory accuracy, working memory span tasks, or task switching. The data suggest that, despite clear motor and balance advantages, dance training does not necessarily transfer to other cognitive abilities in young healthy experts. The findings may suggest that individuals did not self-select into intensive dance regimens because they had naturally different cognitive abilities. In fact, Dancers differed from Non-Dancers only on two tasks, and on both they showed decreased performance: Dancers were slower on the spatial working memory task and had lower *d*′ on the relational memory task.

Interestingly, *d*′ was negatively associated with Dance Central performance and positively associated with PLIC FA across all participants. As Dancers showed lower PLIC FA and better Dance Central performance, it is possible that some aspects of training that favor dance performance come at a cost for relational memory. This unexpected finding needs to be further tested and confirmed in larger samples and with a broader array of tasks to identify, for example, different strategies in Dancers. In addition, slower responses on the working memory task were not associated with dance skill or any brain measures, which may again point to a different strategy used by Dancers. In sum, our findings suggest that dance training benefits mainly motor and balance performance, but may have limited significance for broad cognitive performance in young healthy adults. We note, however, that neural changes related to dance proficiency could have cognitive effects in clinical populations.

### Dance Practice May Alter Microstructure of the Corticospinal Tract

Dancers had decreased FA in the PLIC. PLIC mainly contains fibers of the corticospinal tract, arising in the cortical motor areas and passing downward from the cerebral peduncles to the medulla oblongata. PLIC also contains sensory fibers connecting the thalamus, fibers of optic radiation connecting lower visual centers to the occipital cortex, acoustic fibers connecting the lateral lemniscus to the temporal lobe, and fibers that pass from the occipital and temporal lobes to the nuclei pontis (Mori et al., 2005). The nuclei pontis are part of the pons related to motor activity. Specifically, the nuclei pontis allow modification of movement error correction, and are hence important in learning motor skills. The fibers running through PLIC play an important role in the motor execution, motor learning, visual, and auditory systems, all of which are involved in dance.

Our finding of decreased FA in the dance-trained group is in line with previous findings of lower FA in ballet dancers (Hänggi et al., 2010; Meier et al., 2016) and gymnasts (Huang et al., 2015), as well as of decreased FA in young adults following balance training (Taubert et al., 2010). Therefore, our findings support the notion that training-related reduction in FA in young adults may reflect greater axonal diameter or different patterns of crossing fibers rather than white matter degeneration (Barazany et al., 2009; Alexander et al., 2010). To the best of our knowledge, there are few crossing fibers in the section of PLIC we investigated. Therefore, we argue that greater axon diameter is the more likely explanation of reduced FA in the corticospinal tract of Dancers. Increased axon diameter aids faster transduction of action potentials along fibers of the motor execution and control system by decreasing the internal resistance to passive current flow (Purves et al., 2001). This possibility should be further investigated in dance training intervention studies involving longitudinal measurements of axon diameter distribution (Barazany et al., 2009) and myelin water fraction (Deoni et al., 2012).

### Dancers Show Stronger Activation of the AON

When viewing a dance sequence and instructed to try to imagine doing the dance, both Dancers and Non-Dancers activated the expected AON regions. Involvement of the motor, premotor, sensory, and multisensory cortices is in line with the studies that first demonstrated the AON for hand movement using fMRI in humans (Buccino et al., 2001; Grèzes and Decety, 2001; Iacoboni et al., 2001; Cross et al., 2006).

We cannot exclude the possibility that not all regions were directly related to dance: some may, for example, represent human recognition. However, we argue that the task was sufficiently specific and had sufficient power given that the observed AON activations closely overlapped with fMRI brain activity reported when expert dancers viewed familiar compared to unfamiliar dance stimuli (Calvo-Merino et al., 2005; Cross et al., 2006; Cross and Elizarova, 2014; Kirsch and Cross, 2015).

The activations we observed also overlapped well with brain activity during actual dance activities reported with different neuroimaging tools. A recent electroencephalography study showed brain activity during Laban Movement Analysis in the sensorimotor areas (M1, S1, premotor, and supplementary motor). Activity in the superior temporal gyri was linked to the auditory processing of music, in the basal ganglia to reflecting the processing of metrical movements, and in the thalamus to integration of somatosensory and motor stimuli (Cruz-Garza et al., 2014). Another study used positron emission tomography on amateur dancers who performed dance during the scan. The results showed that movement to a regular, metric rhythm implicated the right putamen, entrainment of dance steps to music was supported by anterior cerebellar vermis, and spatial navigation of leg movement during dance activated the medial superior parietal lobule, reflecting proprioceptive and somatosensory integration (Brown et al., 2006). Finally, a recent fMRI study showed the involvement of bilateral lateral occipital cortex in dance observation in ballet dancers (Di Nota et al., 2016).

Finally, we showed that Dancers activated the AON to a greater extent than Non-Dancers, especially in motor and premotor regions, basal ganglia, and higher visual cortex. There are two non-mutually exclusive mechanisms that could explain increased activity in Dancers: strategy, and differences in neural processing. First, watching someone perform a sequence to memorize and repeat it is a routine part of training in expert dancers. For example, professional dancers are known to have a superior ability to remember moves with high accuracy using different techniques like combining certain moves into chunks (Starkes et al., 1990), similar to chunking working memory strategies (Tulving and Craik, 2000). Proficiency in this movement learning likely relies on creating successful representations of information to be chunked. We propose that Dancers’ greater ability to activate the AON related to dance would aid the movement learning process. This is consistent with the increasing magnitude of AON activation in lateral occipital cortex during dance visualization during 8-months of learning a choreography in professional ballet dancers (Di Nota et al., 2016) and with the finding that dancers consistently show greater AON activity to familiar dance stimuli (Calvo-Merino et al., 2005; Cross et al., 2006; Cross and Elizarova, 2014; Kirsch and Cross, 2015). Although we were not able to test performance on the dance-viewing task, the superior scores of Dancers compared to Non-Dancers in Dance Central support the possibility that Dancers are better-skilled in recruiting the task-relevant neural systems.

Second, it may be that Dancers process the dance information in a different way than Non-Dancers such that their motor learning system is altered in its efficiency and organization as a result of training. Efficiency is often understood as less effortful processing, and therefore would most likely be associated with smaller BOLD signal changes (Burzynska et al., 2013). However, our task was not parametric (did not involve different levels of task difficulty), nor did we assess the quality of dance processing by the ability to repeat the sequences. In addition, fMRI provides no direct information about neurotransmission or neuromodulation. Therefore, in the current study we were not able to resolve the role of neural efficiency in BOLD magnitude differences. However, we were able to assess the functional organization of the motor network and the AON by studying resting-state FC within these networks.

### Dancers Show Altered FC of the AON and the Motor Learning Networks

We observed group differences in FC within brain regions where Dancers activated the AON to a greater extent than Non-Dancers. We found increased FC between the thalamus and the frontal pole in Dancers compared to Non-Dancers. The finding suggests that the part of the AON that was activated to a greater extent by Dancers is differentially connected to the prefrontal cortex. It is possible that better top-down modulation of the AON from the prefrontal cortex via enhanced thalamo-cortical connections may lead to greater recruitment of this network in Dancers. This notion is supported by our finding of a positive correlation of enhanced thalamo-frontal FC and objective measure of dance skill. Therefore, groups differences in FC may reflect a cognitive aspect of dance training, as thalamo-frontal projections are involved in several cognitive functions, such as rule-based learning, attention, working memory and language (Bonelli and Cummings, 2007; Oh et al., 2009). Importantly, thalamo-frontal FC was shown to be critical in top-down cognitive functions (Monti et al., 2015).

A similar investigation within the general (i.e., common to all participants) AON yielded more complex results. Dancers showed both enhanced connections of the AON to regions involved in somatosensory association (supramarginal gyrus) and sensory interpretation and body image (inferior parietal lobule), as well as attenuated connections with face and body recognition (fusiform gyrus), visual regions (occipital pole), and visual perception of the human body and body parts (lateral occipitotemporal cortex, known also as the extrastriate body area; Downing et al., 2001). The connections between cortex and basal ganglia involve a complex system of excitatory and inhibitory connections. With fMRI we cannot precisely interpret the meaning of attenuated or enhanced FC. However, correlations with balance and dance skill performance confirmed that lower or greater FC in Dancers was associated with better performance outcomes, suggesting that these FC differences were relevant for dance training.

Similarly, some regions from a broadly defined motor learning network (Hardwick et al., 2013) were differentially connected with other brain regions in Dancers and Non-Dancers. These included regions involved in sensory and motor functions (postcentral and precentral gyri), face and body recognition (fusiform gyrus), as well as regions involved in motor coordination, visuospatial processing and imagery (precuneus), and executive function and control (inferior frontal gyrus). Again, at the individual level, the strength of these connections correlated with dance skill. Importantly, the correlation of some FC in the motor learning network with PLIC FA suggests that structural and functional brain characteristics differentiating Dancers from Non-Dancers are related and behaviorally relevant. This is the first evidence for structure-function relationships of the effects of dance training on the human brain.

Finally, the mix of enhanced and attenuated FC of the motor learning network in Dancers is consistent with previous short-term motor skill training interventions, which reported both increases and decreases in FC (Albert et al., 2011; Taubert et al., 2011; Vahdat et al., 2011; Yoo et al., 2013). However, there may be differences between short-term (days–months) and long-term (years, as in our study) effects of whole-body training on brain FC. Future longitudinal studies following dance skill acquisition in the first days and weeks, to months and years are necessary to fully understand the effects of dance training on the human brain.

### Decreases in Brain Volume Are Not a General Correlate of Dance Training

We found no regions where Dancers had thicker cortex or larger subcortical volumes than Non-Dancers. The finding rules out our third hypothesis that long-term, versatile, expert dance training follows the pattern of structural brain plasticity reported for other motor tasks (Thomas and Baker, 2013). This may be surprising given the body of evidence of increased cortical volumes as a result of motor training interventions or in experts compared with novices (Boyke et al., 2008; Thomas and Baker, 2013). For example, expert- relative to novice-golfers had increased gray matter volume in the premotor and posterior parietal cortex, (Jäncke et al., 2009), 6-week juggling training resulted in bilateral expansion in the mid-temporal area and in the left posterior intra-parietal sulcus, regions associated with bimanual arm movement, grasping, and visual tracking (Taubert et al., 2010), and expert dancers and musicians had thicker superior temporal cortex than controls (Karpati et al., 2017).

However, dance training, is more multidimensional than specific motor training in juggling or golf. It involves whole body movement, memorizing complex sequences, and synchronization with music and other dancers, which could result in weaker, more diffuse rather than focal increases in volume in motor regions. Importantly, modern dance training is more versatile in style than ballet training, which may lead to weaker focal effects than observed by Meier et al. (2016). The fact that Karpati et al. (2017) found greater cortical thickness in dancers and musicians could be due to the inclusion of men in their sample. The gender-specific patterns of training-related neuroadaptation or plasticity require further investigation. Another possibility is that the initial increases in local gray matter volume as a result of days/weeks of training are no longer detectable after years. This possibility is supported by evidence from prior work, which indicated that volume increases in the motor cortex after hand motor training persist during first 4 weeks of training, but disappear thereafter (Wenger et al., 2016).

Interestingly, we observed a trend-level cortical thinning in Dancers in the sensorimotor cortex, overlapping with the AON (precentral and postcentral gyri). Our findings give only weak support to the notion that expert dance training is related to neuroplasticity manifested by lesser brain volume (Hänggi et al., 2010), given that our sample was larger and matched for BMI, and we used more sensitive surface-based cortical measurements. We speculate that smaller brain volume in ballet dancers reported by Hänggi et al. (2010) is due to a combination of low BMI, restricted diet, and rigorous ballet-only training. These factors may modulate the effects of training on brain structure and function in the developing brain. For example, a certain percentage of body fat is necessary to maintain hormonal homeostasis and the related neuroprotective effects of estrogen (Nascimento et al., 2012). Therefore, constantly low body fat may affect the brain directly or indirectly via hormone regulation. There is emerging evidence of how too-low (Seitz et al., 2014) or too-high BMI (e.g., Mueller et al., 2011) may negatively impact the central nervous system and introduce variance in the data not related to dance experience and skill. Rigorous physical practice and well-defined movement vocabulary in ballet (Kersley and Sinclair, 1981) could result in specification and pruning within certain brain networks that may not be observed in versatile-trained modern dancers in our study (Reynolds and McCormick, 2003). Interestingly, musical training has been connected with increases in brain volume in regions related to higher cognitive functioning, but decreases in volume of sensorimotor regions (James et al., 2013). Our observation of trend-level reduction of cortical thickness in sensorimotor regions could be related to neural pruning leading to greater motor efficiency and automation (James et al., 2013), though the effect may be weaker than in the hand region of musicians. Together, when matching for age, education, and BMI, we found weak evidence for structural cortical neuroadaptations related to dance proficiency in healthy young adults.

### Limitations

We have to highlight several possible limitations of our study. First, the cross-sectional design does not allow for studying causal effects of dance training on brain structure and function. Factors not related to dance training, such as innate predispositions or genetic background, lifestyle, or baseline brain structure and function may contribute to some of the observed group differences. We attempted to minimize these limitations by implementing strict study inclusion criteria and matching groups by age, BMI, and education. Second, we have focused on the effects of expert long-term versatile dance training, including multiple dance styles (e.g., ballet, modern, jazz, and hip-hop). Therefore, our findings may not be specific to dance but may reflect broader sensorimotor and aesthetic training (Meier et al., 2016). Future multimodal MRI studies should compare the effects of long-term training of different dance styles with active control groups to identify the most effective dance routines for altering brain structure and function. Third, we only recruited female subjects to minimize sources of additional between-sex variance. Future studies should extend our results to male dancers to assess generalizability of the results acquired in female dancers. Finally, several other factors such as menstrual cycle (Lisofsky et al., 2015), circadian cycle (Schmidt et al., 2012) or seasonal changes (Meyer et al., 2016) may influence brain structure and function. Therefore, these factors should be assessed in future studies on larger samples. Furthermore, certain effects we observed were weak after correcting for multiple comparisons. Future studies need to include larger samples. However, we wanted to highlight the importance of evidence in support of the hypotheses in addition to negative findings (Gorgolewski and Poldrack, 2016).

## Conclusion

We showed that Dancers’ brains differed from Non-Dancers’ at both functional- and structural-levels. Most of the group differences were skill-relevant and correlated with objective laboratory measures of dance skill and balance. Our results are promising in that long-term, versatile, combined motor and coordination training may induce neural alterations that support performance demands. Dance training could be used instead of, or in addition to neurostimulation techniques to modulate the brain function and structure to optimize skill acquisition and motor performance (Reis et al., 2009; Galea et al., 2011). This would be in line with the theoretical framework of increasing motor and neural reserve to optimize performance and postpone age- or disease-related declines in function (Heuninckx et al., 2008; Palmer et al., 2009). However, the plastic potential of the brain in response to dance training in young healthy adults needs to be demonstrated in older adults, clinical populations, and males. In sum, our multimodal neuroimaging results tap into neural mechanisms underlying physical and functional benefits reported in epidemiological studies (Kattenstroth et al., 2013), as well as protective effects on cognition in older dancers (Ballesteros et al., 2015; Adam et al., 2016; Burzynska et al., 2017).

## Author Contributions

AB and AFK designed the study. AFK provided funding and equipment. AB and AMK designed tools and collected data. AB analyzed cognitive, dance skill, and MRI structural data. KF and BT analyzed functional MRI data. AB, KF, and BT wrote the manuscript.

## Conflict of Interest Statement

The authors declare that the research was conducted in the absence of any commercial or financial relationships that could be construed as a potential conflict of interest.

## Footnotes

1 http://surfer.nmr.mgh.harvard.edu/

2 http://www.nitrc.org/projects/conn/

3 www.nitrc.org/projects/artifact_detect

4 http://marsbar.sourceforget.net
