# Supplementary material for: The Dancing Brain: Structural and Functional Signatures of Expert Dance Training
Source: Front Hum Neurosci. 2017 Nov 27;11:566. doi: 10.3389/fnhum.2017.00566 (PMC5711858; doi:10.3389/fnhum.2017.00566)
Supplement: Supplementary file 1 [file Data_Sheet_1.docx]

**The dancing brain: Structural and functional signatures of professional dance training.**

**Supplementary Material I.** Detailed inclusion and exclusion criteria

| Exclusion criteria |  |  |
| --- | --- | --- |
| General | Dancers | Non-Dancers |
| Taking any anti-depressants, anti-anxiety, anti-psychotic meds now or in the last 2 months  Any major medical or mental conditions, mobility problems, ADHD, ADD, depression, or any other psychological or psychiatric condition  Vision problems other than corrected by lenses  Hearing problems  Epilepsy  Reading disability  Head injury with loss of consciousness  Claustrophobia  Metals in your body (as a result of injury with a metal object, accident, or surgery), tissue expanders  A pacemaker or any other implant (aortic clips, insulin pump)  Pregnancy or chance thereof  BMI <18 or >25  Any history of anorexia  Any meds taken to help reduce body weight  Lack of fluency in English | Only training ballet  < 10/week spend on dancing, including technique, ballet, modern, ballroom, jazz, hip-hop, choreography, performing, but also classes such as yoga/pilates  <3 years of intense dance training history (>10 hours/week) | “Do you consider yourself a dancer?” answered with “yes”  Are you currently training dancing? Answer “yes”  > 10/week spend on dancing, including technique, ballet, modern, ballroom, jazz, hip-hop, choreography, performing, but also classes such as yoga/pilates  >3 years of intense dance training history (>10 hours/week), especially in the last 10 years  > than two years of current casual dance/yoga, etc. class experience  Professional training/participation in related activities such as ballet, ballroom dancing, hip-hop, jazz, capoeira, yoga, ice skating, acrobatics, gymnastics, slacklining  Regular use/high proficiency in dance video games |
| Inclusion criteria |  |  |
| General | Dancers | Non-Dancers |
| Female  Age 18-32  18 <BMI <25 | “Do you consider yourself a dancer?” answered with “yes”  Are you currently training dancing? Answer ”yes”  > 10/week spend on dancing, including technique, ballet, modern, ballroom, jazz, hip-hop, choreography, performing, but also classes such as yoga/pilates  >3 years of intense dance training history (>10 hours/week) | “Do you consider yourself a dancer?” answered with “no”  Are you currently training dancing? Answer ”no”  < 10/week spend on dancing, including technique, ballet, modern, ballroom, jazz, hip-hop, choreography, performing, but also classes such as yoga/pilates  <3 years of intense dance training history (>10 hours/week) in the last 10 years |

**Supplementary Material II.** Subject flow. NP: neuropsychological testing.


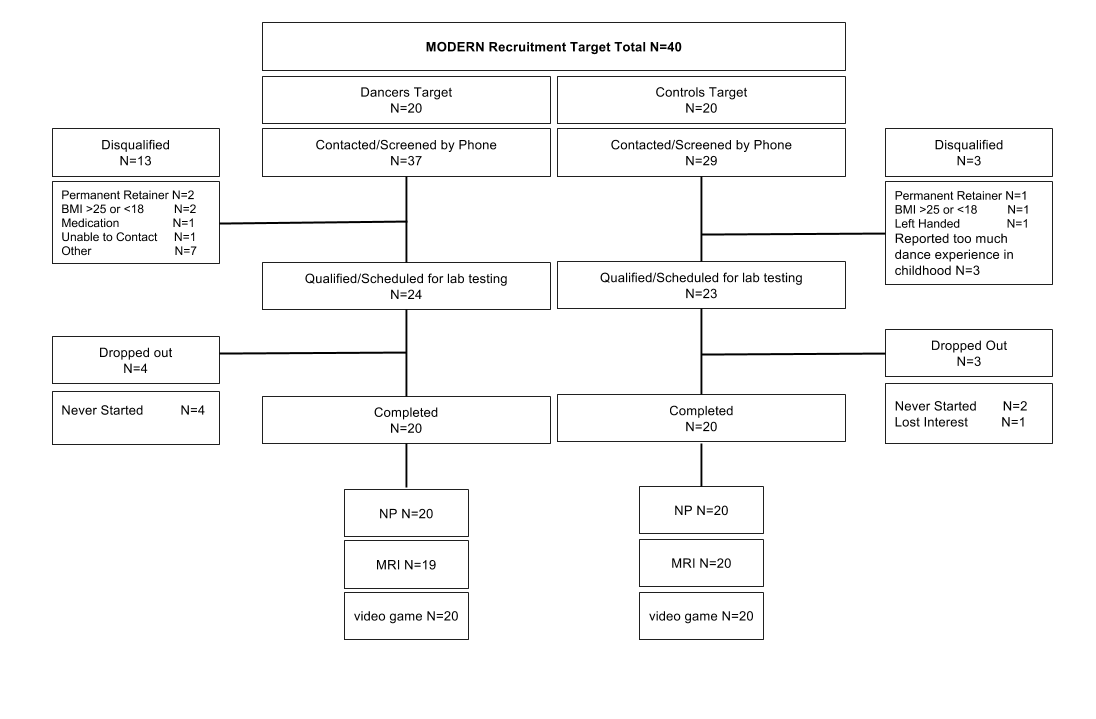


**Supplementary Material III.** Cognitive battery and the result of dimensionality reduction with PCA.

| Task | Construct | Description | Administration | Source | Fluid intelligence | Perceptual speed |
| --- | --- | --- | --- | --- | --- | --- |
| Matrix reasoning | Fluid intelligence | Select pattern that best completes the missing cell in a matrix | Computer-based | [(Raven 1962)](#h.3znysh7) | .819 | – |
| Shipley abstraction |  | Determine the letters, words, or numbers that best complete a progressive sequence | Paper-pencil | [(Zachary 1986)](#h.3dy6vkm) | .716 | – |
| Letter sets |  | Identify which of five groups of letters is different from the others | Computer-based | [(Ekstrom et al. 1976)](#h.1fob9te) | .735 | - |
| Spatial relations | Spatial reasoning | Determine which three dimensional object could be constructed by folding the two dimensional object | Computer-based | [(Bennett et al. 1997)](#h.30j0zll) | .831 | – |
| Paper folding |  | Determine the pattern of holes that would result from a sequence of folds and a punch through folded paper | Computer-based | [(Ekstrom et al. 1976)](#h.1fob9te) | .670 | – |
| Form boards |  | Determine shapes needed to fill in a space | Computer-based | [(Ekstrom et al. 1976)](#h.1fob9te) | .661 | – |
| Digit symbol | Perceptual speed | Use a code table to write the correct symbol below each digit | Paper-pencil | [(Wechsler 1997)](#h.tyjcwt) | – | .684 |
| Letter & pattern comparison |  | Same or different comparison of pairs of letter strings/patterns | Paper-pencil | [(Salthouse and Babcock 1991)](#h.2et92p0) | –  - | .843  .770 |

Columns 6–9: Standardized component loadings from a 4-factor PCA extraction. For clarity, only loadings above 0.30 are displayed. Rotation method: varimax with Kaiser normalization. Rotation converged in 6 iterations. Pairwise exclusion was performed.

**Spatial Working Memory**

Participants completed a version of a spatial working memory paradigm previously used in our lab (Erickson et al. 2009, 2011; Baniqued et al. 2014). In this task, individuals were required to remember the location of dots on a computer screen. In the encoding phase of a trial, individuals studied the locations of two or three dots for 500 msec, or four dots for 1 sec. This was followed by a 3000-msec delay period where the fixation cross was present, after which one probe red dot appeared on the screen and participants were instructed to indicate yes/no as to whether the probe dot occupied the same space as one of the dots in the encoding phase; the red probe dot was displayed for 2000 msec, during which time the participants had to respond. The trials were separated with a 1000-msec inter-trial interval. Following 12 practice trials (6 match, 6 non-match), participants completed the actual experiment that contained 40 trials (20 match and 20 non-match) for each set size, presented in an intermixed fashion.

**Relational Memory**

We used a relational memory task where individuals had to remember pairs of faces and scenes (Hannula et al. 2006, 2007; Monti et al. 2013; Walker et al. 2014). The task was divided into three separate runs, with 24 encoding and 24 recognition trials in each run; the encoding and recognition phases were separated by a 20- sec rest period. Encoding and recognition trials consisted of the presentation of a scene for 2000 msec followed by a face overlaid on the scene for an additional 2000 msec. A fixation cross was displayed during the inter-trial interval which was jittered and ranged from 2000 to 12000 msec. Participants completed a practice session before proceeding with the task. On each encoding trial, participants made a yes/no judgment indicating whether the individual depicted “fit” with the scene; this was an arbitrary decision to elicit deep encoding. At recognition two trial types were presented, “intact” face–scene pairs, which were the identical face–scene combinations presented during encoding, and “re-pair” trials, created by recombining a previously displayed face and scene that were not shown together at encoding. Hence, all stimuli were equally familiar at recognition, and the task had to be completed via relational memory. Participants made a yes/no judgment as to whether the pair displayed was an exact match of a pair shown at encoding, with 12 trials from each trial type composing the recognition phase of a run.

**Span Tasks**

The digit span task measures verbal working memory and it tests how many items (numbers) can be stored and repeated, in a sequential order (Wechsler 1997). The participants were asked to remember a series of numbers in two conditions - forward and backward.  In the forward condition, the experimenter read a sequence of three digits and the participant was asked to recite the sequence in the same order. With each string of numbers recalled correctly, we increased difficulty by adding a number to the sequence to a maximum of nine. The task was terminated after the participant could not recall correctly the full sequence on the second attempt. In the backward condition, participants were asked to recall the series of digits in the reverse order, with the minimum of two and maximum sequence length of eight.

**Trail Making Task**

This task measures visual attention and task switching. In Trail Making Tests A and B (Reitan 1992) there are 25 circles distributed on a page with numbers (1-25) in each circle for trails A, or numbers (1-13) and letters (A-L) for trails B. The participant’s task is to accurately draw lines connecting the circles in ascending order for trails A (1,2,3, etc.), and alternating between numbers and letters in ascending, alphabetical order for trails B (1-A-2-B, etc.). Participants are asked to perform this task as quickly as possible and without lifting their pencil. The test administer then takes amount of time it took the participant to complete the task as the dependent variable.  The outcome variables were the time taken to complete task A, task B, as well as the difference between the two (B-A), which reflects the task switching “cost”.

**Supplementary Material IV.** Voxel-wise analysis of the cortical thickness


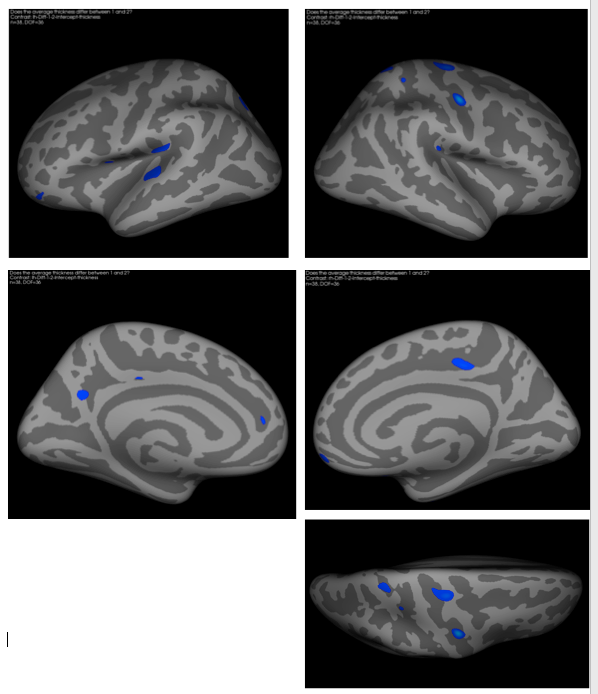


SM Fig. 14. Dancers showed a trend towards a thinner cortex than Non-Dancers, especially in the R precentral cortex, p<.01, uncorrected.

**Supplementary Material V.** Whole-white matter TBSS results of DTI analysis


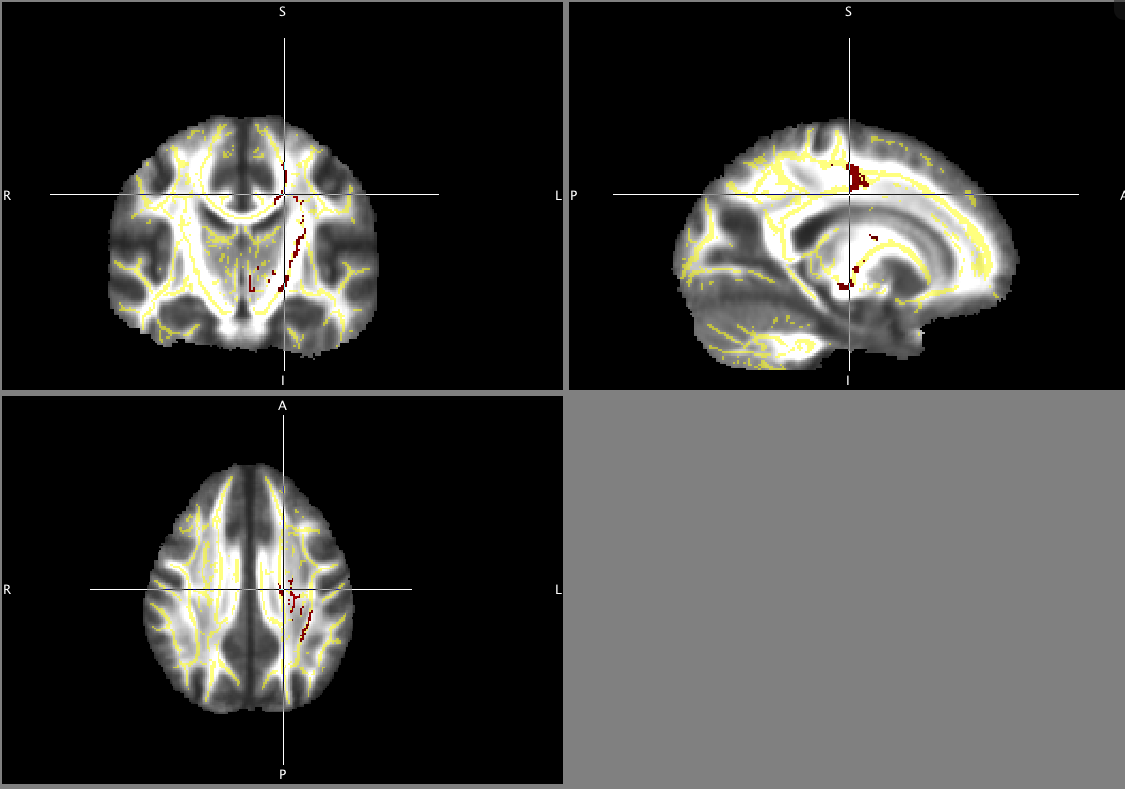


SM Fig. 13. Whole-brain randomize 5000 permutations TFCE comparison between Dancers and Non-Dancers (in TBSS). Dancers showed a trend (p>.10) towards lower FA (in red) in the (mainly left) corticospinal tract, including sections of the primary motor/premotor cortex, the body corpus callosum, and the posterior section of the superior longitudinal fasciculus.

**Supplementary Material VI.** fMRI task results for additional contrasts. All images are thresholded at z>2.3.


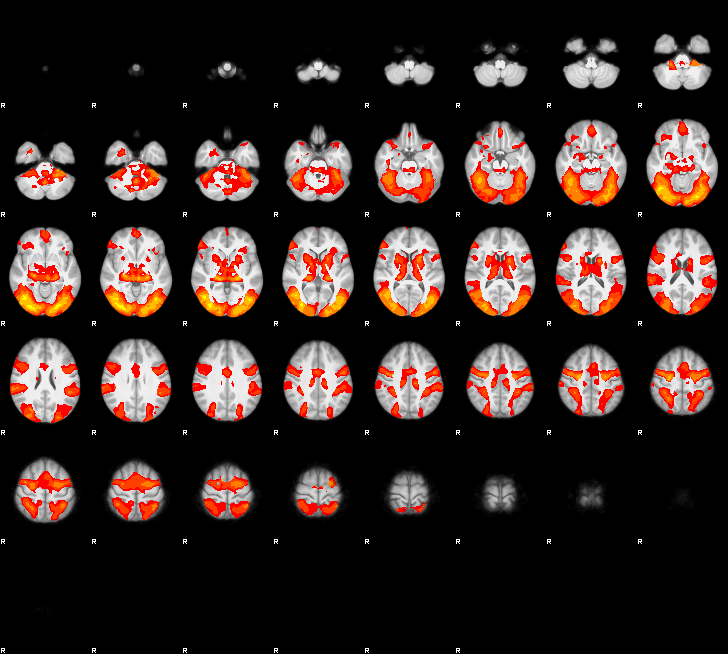


SM Figure 1. BOLD signal increases for the Dance > Fixation (baseline), Dancers only (n = 19).


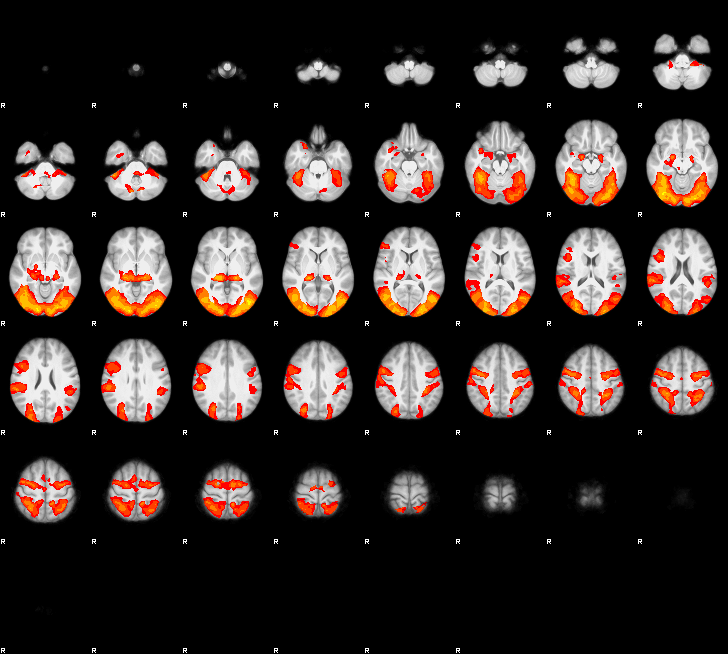


SM Figure 2. BOLD signal increases for the Dance > Fixation (baseline), Non-Dancers only (n = 20).


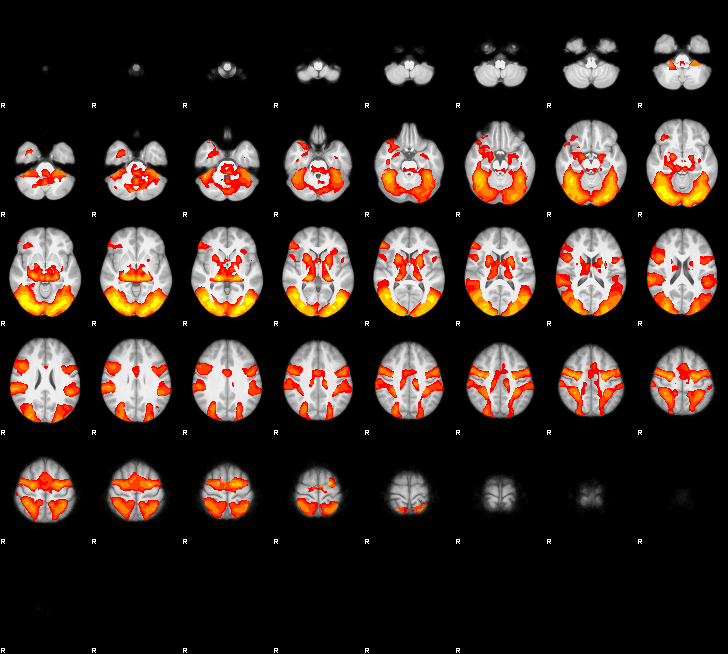


SM Figure 3. BOLD signal increases for the Dance > Fixation (baseline), full sample combined (n = 39).


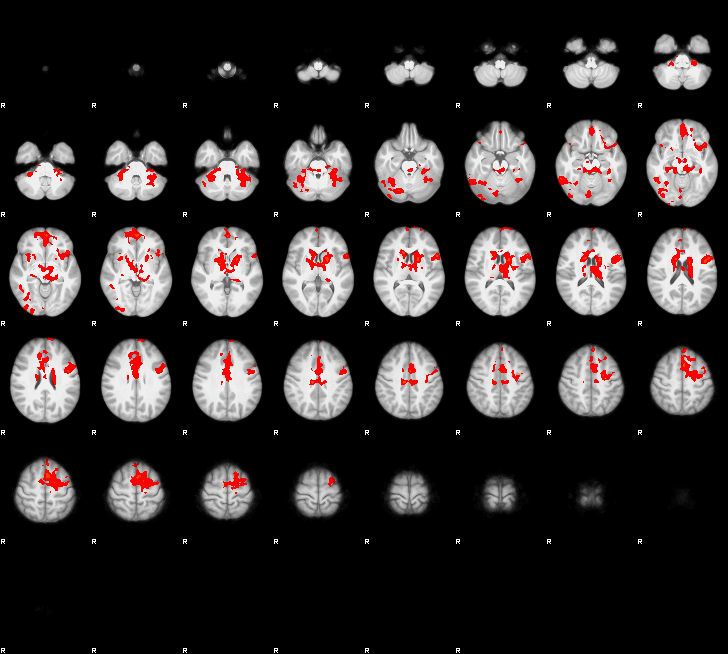


SM Figure 4. BOLD signal for the Dance > Fixation, Dancers > Non-Dancers. Note: For the opposite contrast (Non-Dancers > Dancers) yielded no significant regions.


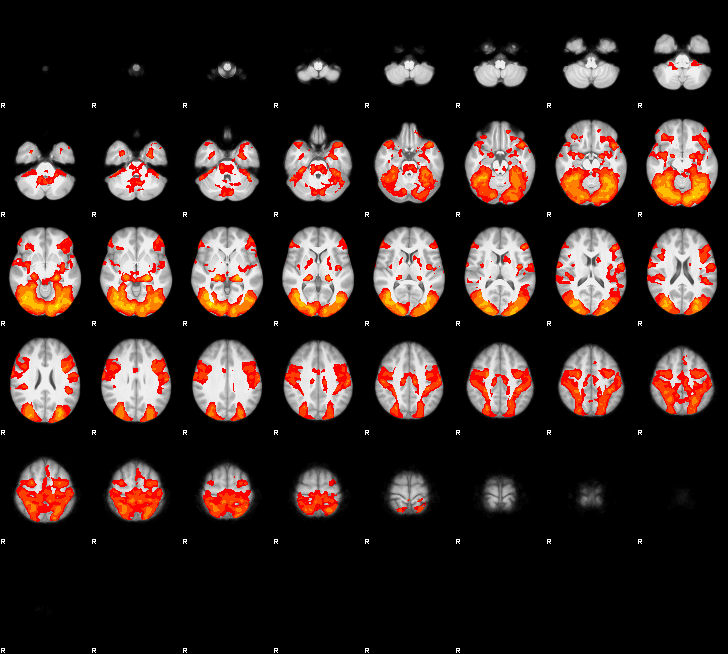


SM Figure 5. BOLD signal increases for the Control > Fixation, Dancers only (n = 19).


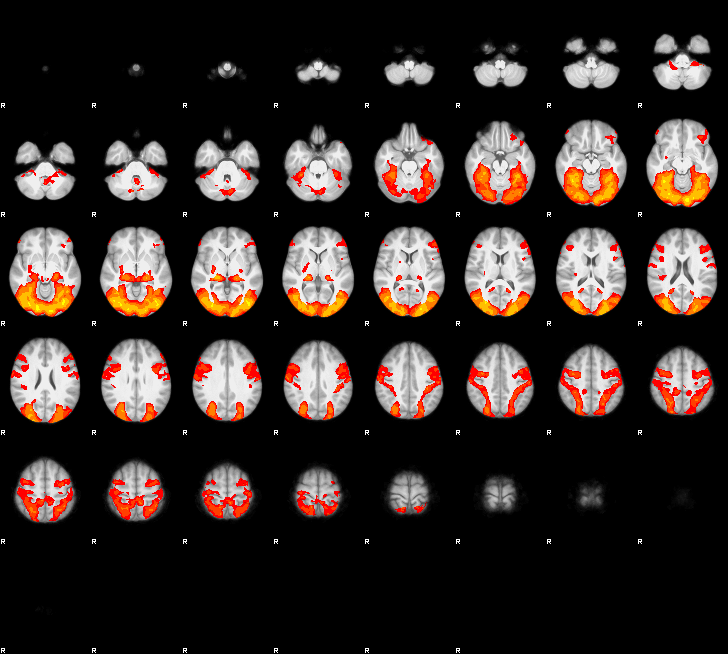


SM Figure 6. BOLD signal increases for the Control > Fixation, Non-Dancers only (n = 20).


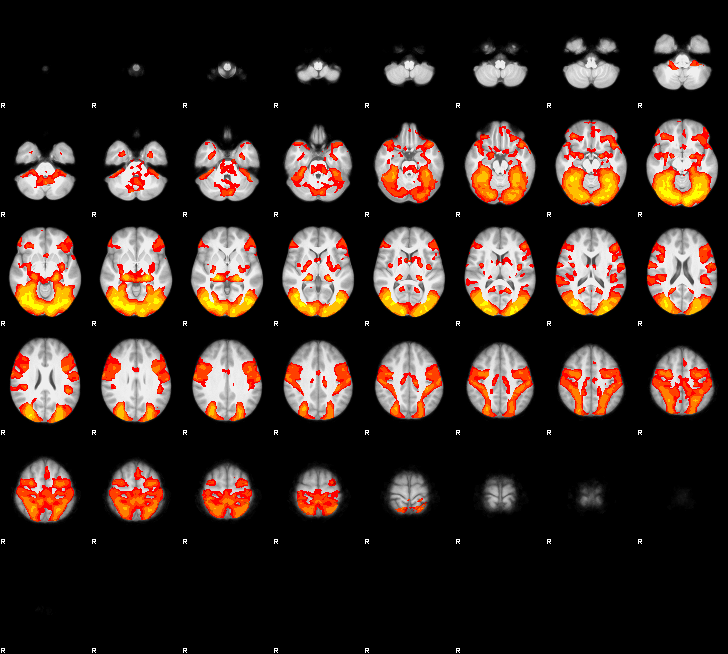


SM Figure 7. BOLD signal increases for the Control condition > Fixation, full sample combined (n = 39).


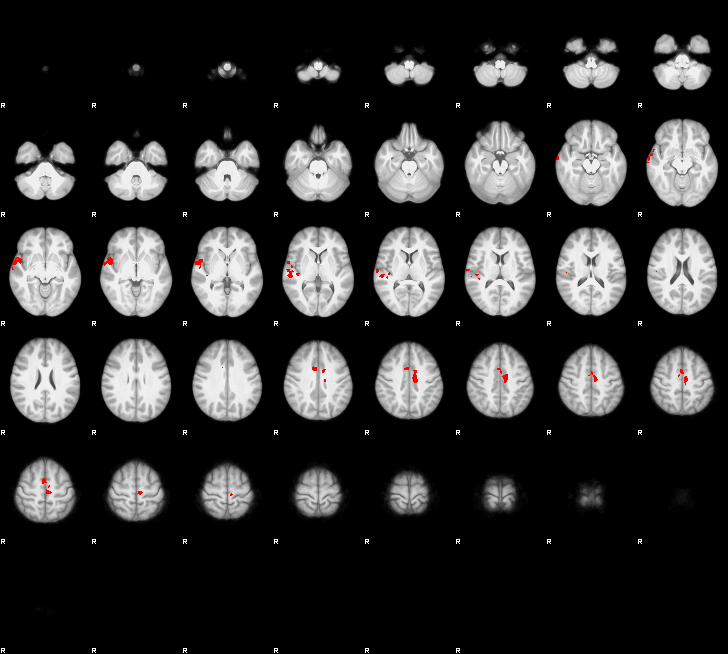


SM Figure 8. BOLD signal increases for the Control > Fixation, Dancers > Non-Dancers (n = 39). Note: The opposite contrast (Non-Dancers > Dancers) yielded no significant differences.


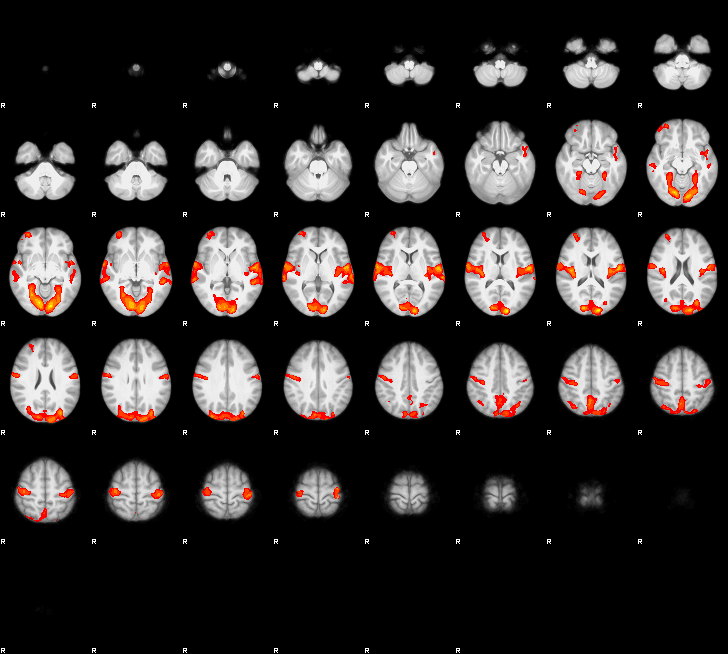


SM Figure 9. BOLD signal increases for the Control > Dance condition, Dancers only (n = 19).


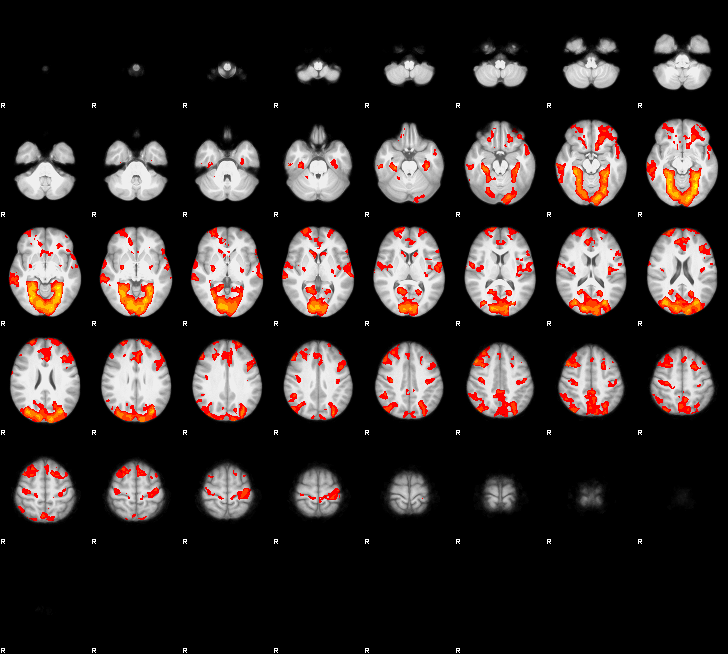


SM Figure 10. BOLD signal increases for the Control > Dance condition, Non-Dancers only (n = 20).


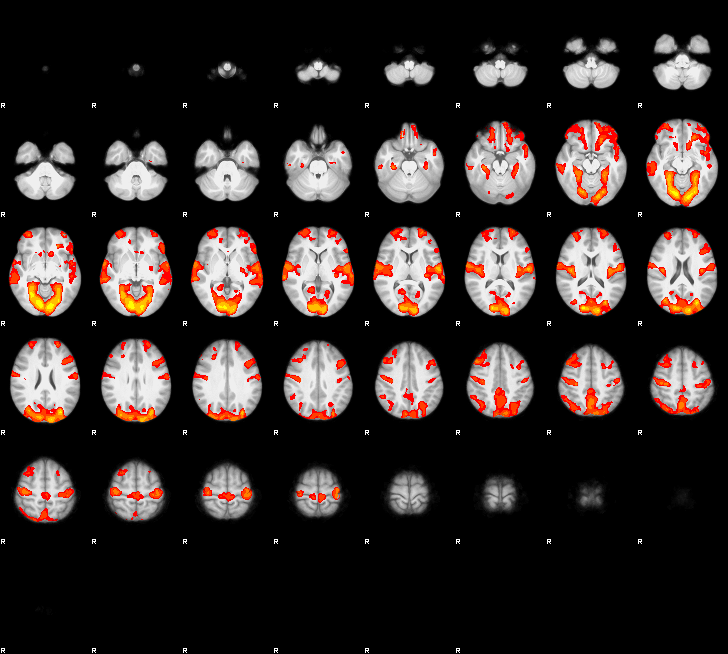


SM Figure 11. BOLD signal increases for the Control > Dance condition, for the full sample (n = 39).


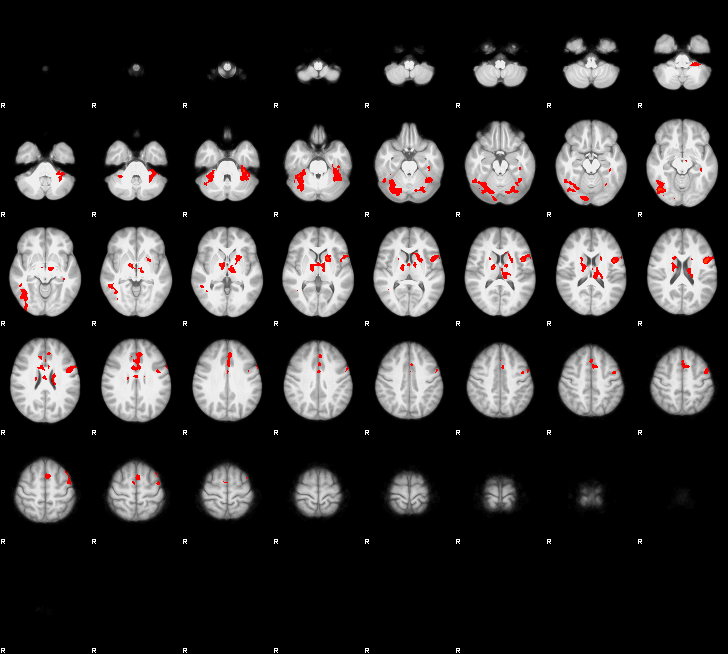


SM Figure 12. BOLD signal increases for the Control > Dance condition, Non-Dancers > Dancers (n = 39). Note: For the opposite contrast (dancer > Non-dancer), there were no significant regions.

**Supplementary Material VII.** Seed coordinates for mirror system and motor learning network.

| Seed region (all radius 4mm) | MNI coordinates | | |
| --- | --- | --- | --- |
|  | *x* | *y* | *z* |
| *Mirror system: Dancers > Non-Dancers* | | | |
| (1) R thalamus | 14 | -8 | 10 |
| R pallidum | -22 | 10 | -2 |
| L caudate | -14 | 16 | -10 |
| L cerebral peduncle | -1 | -10 | -8 |
| L thalamus | -10 | -14 | 2 |
|  | -10 | -22 | 14 |
| L insula/prefrontal/precentral | -44 | 6 | 14 |
| L precuneus | -4 | 12 | 56 |
|  | -4 | 34 | 30 |
| R V1 | 44 | -70 | -12 |
|  | 12 | -88 | -16 |
|  | 50 | -44 | -4 |
| L precentral | -56 | 4 | 38 |
|  | -56 | 10 | 22 |
|  | -46 | 0 | 46 |
| R supp. motor | 4 | -2 | 62 |
| *Mirror system: Dancers & Non-Dancers* |  |  |  |
| (2) L V5 | -48 | -78 | 8 |
| (3) L premotor | -12 | -6 | 66 |
| (4) R SPL/S1 | 34 | -38 | 50 |
| (5) L SPL/S1 (+) | -32 | -42 | -50 |
| R V4 | 50 | -78 | 8 |
| R premotor (+) | 22 | -8 | 62 |
| R supp. motor (+) | 6 | 2 | 60 |
| R putamen | 24 | 10 | 6 |
| L putamen (+) | -24 | 8 | 6 |
| *Motor learning network* |  |  |  |
| (6) L SPL | -30 | -56 | 64 |
| (7) R M1 | 40 | -20 | 54 |
| (8) R putamen | 26 | 0 | 2 |
| L dorsal premotor | -32 | -12 | 60 |
| L supp. motor | 0 | -2 | 56 |
| R supp. motor | 2 | 8 | 52 |
| L thalamus | -12 | -20 | 10 |
| L M1 | -38 | -24 | 58 |
| R S1 | 32 | -24 | 62 |
| L putamen | -26 | 4 | 4 |

**Numbers 1–8:** reference to seed regions with significant group FC differences as presented in Table 2 and Figure 1. (+) regions added to ensure the symmetry of the investigated regions. SPL: superior parietal lobule, S1: primary somatosensory cortex, M1: primary motor cortex, V1: primary visual cortex.

**Supplementary Material VIII.** Functional connectivity and head motion.

In order to test whether the two groups differed on the motion magnitude or pattern during the scanning, we calculated *motion fingerprint* – mean total displacement and scan-to-scan displacement – for each participant (Wilke 2012). This method provides an individualized and comprehensive indicator of head motion taking into account individual differences in brain anatomy.

We compared the head motion parameters with the aim to investigate whether observed FC differences between groups were not driven by group differences in head motion. There were no significant differences between Dancers and Non-Dancers in mean total displacement (M_Dancers_ = 0.59, M_Non-Dancers_ = 0.58, *p* = .899), scan-to-scan displacement (M_Dancers_ = 0.08, M_Non-Dancers_ = 0.09; *p* = .577) and the number of outlier scans (M_Dancers_ = 9.81, M_Non-Dancers_ = 8.71, *p* = .617). These results showed that there is no evidence that the observed group differences in connectivity were driven by group differences in subjects’ head motion.

**Supplementary Material IX.** Scatterplots for selected correlations.


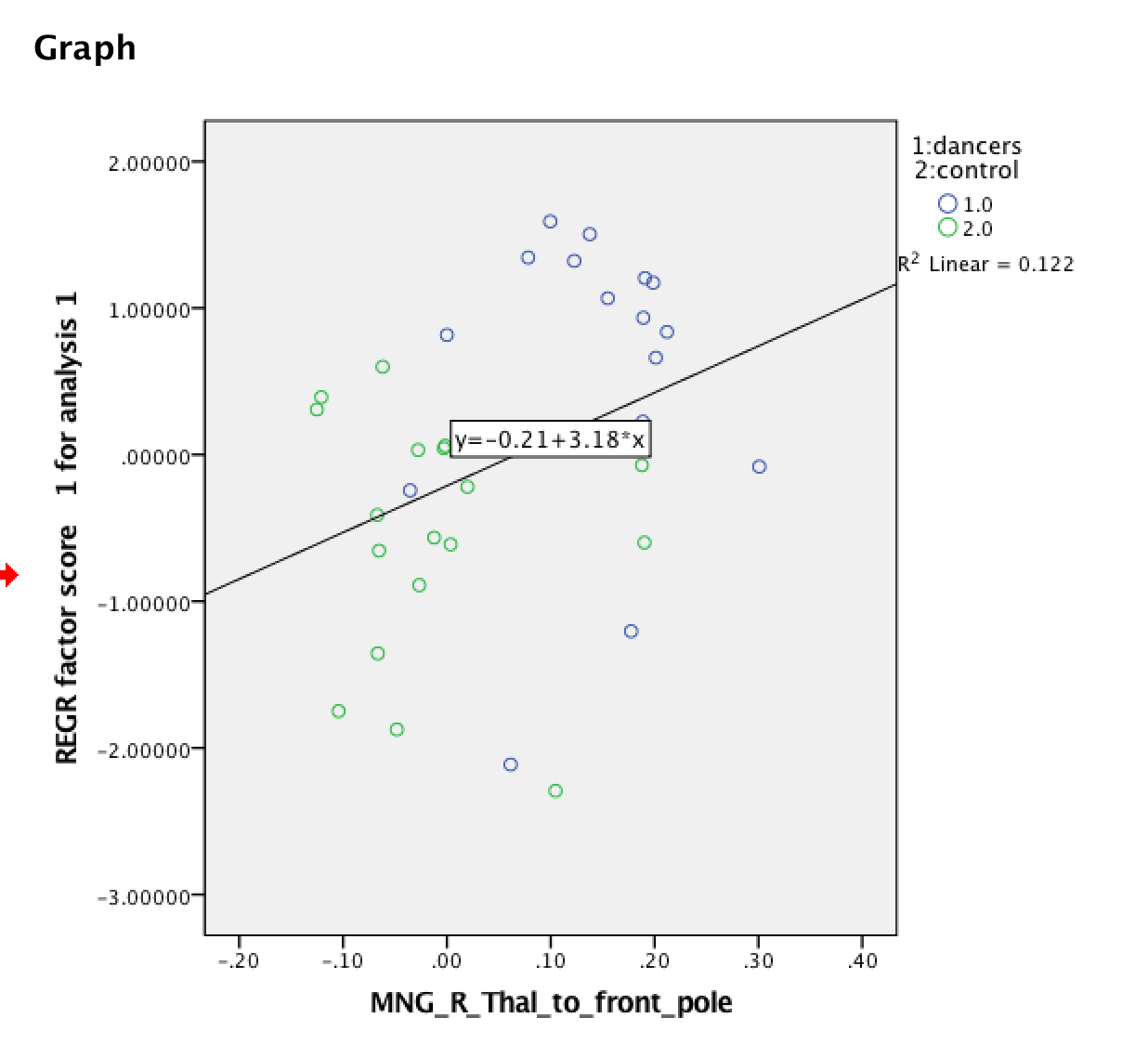


Scatterplot 1: Correlation between FC R thalamus to the R frontal pole (seeds based on Dancers>Non-Dancers contrast of the AON system), and the PCA score for the Dance Video game (% correct of 6 trials). N=36 (whole sample)


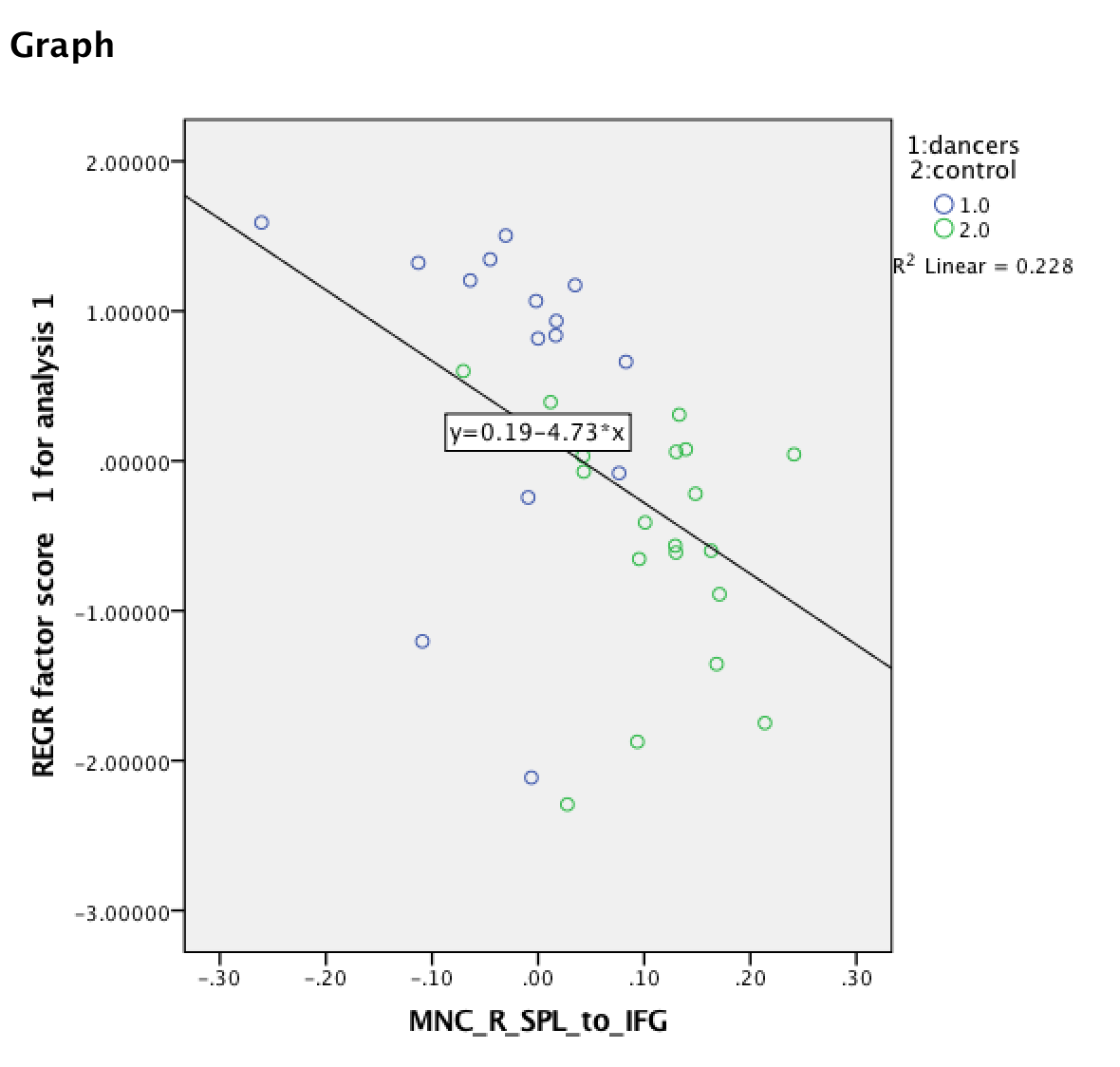


Scatterplot 2: Correlation between FC R SPL to the R IFG (seeds based on Dancers & Non-Dancers contrast of the AON system), and the PCA score for the Dance Video game (% correct of 6 trials). N=36 (whole sample)


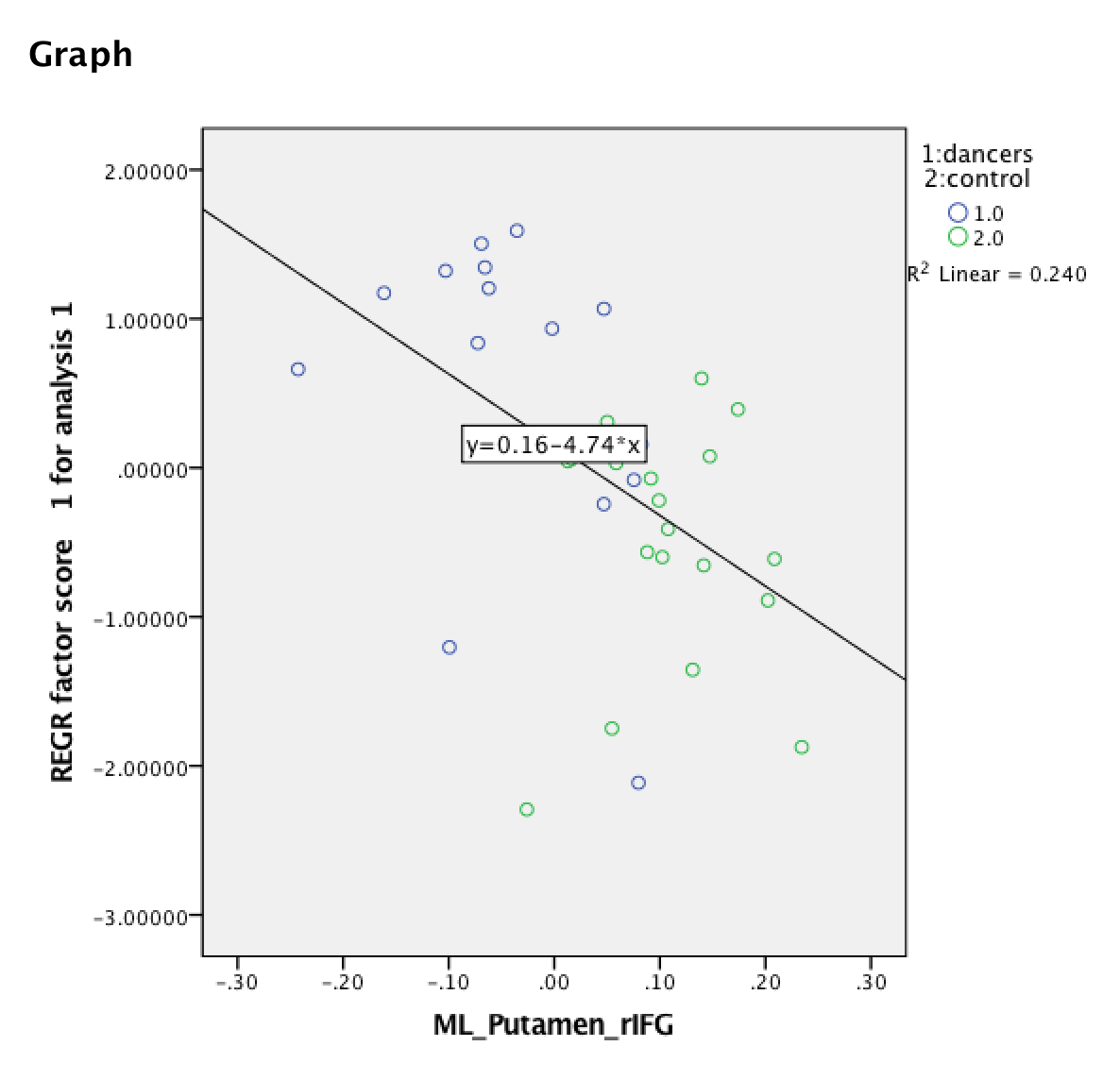


Scatterplot 3: Correlation between FC R Putamen to the R IFG (seeds based on the Motor Learning network), and the PCA score for the Dance Video game (% correct of 6 trials). N=35 (whole sample)


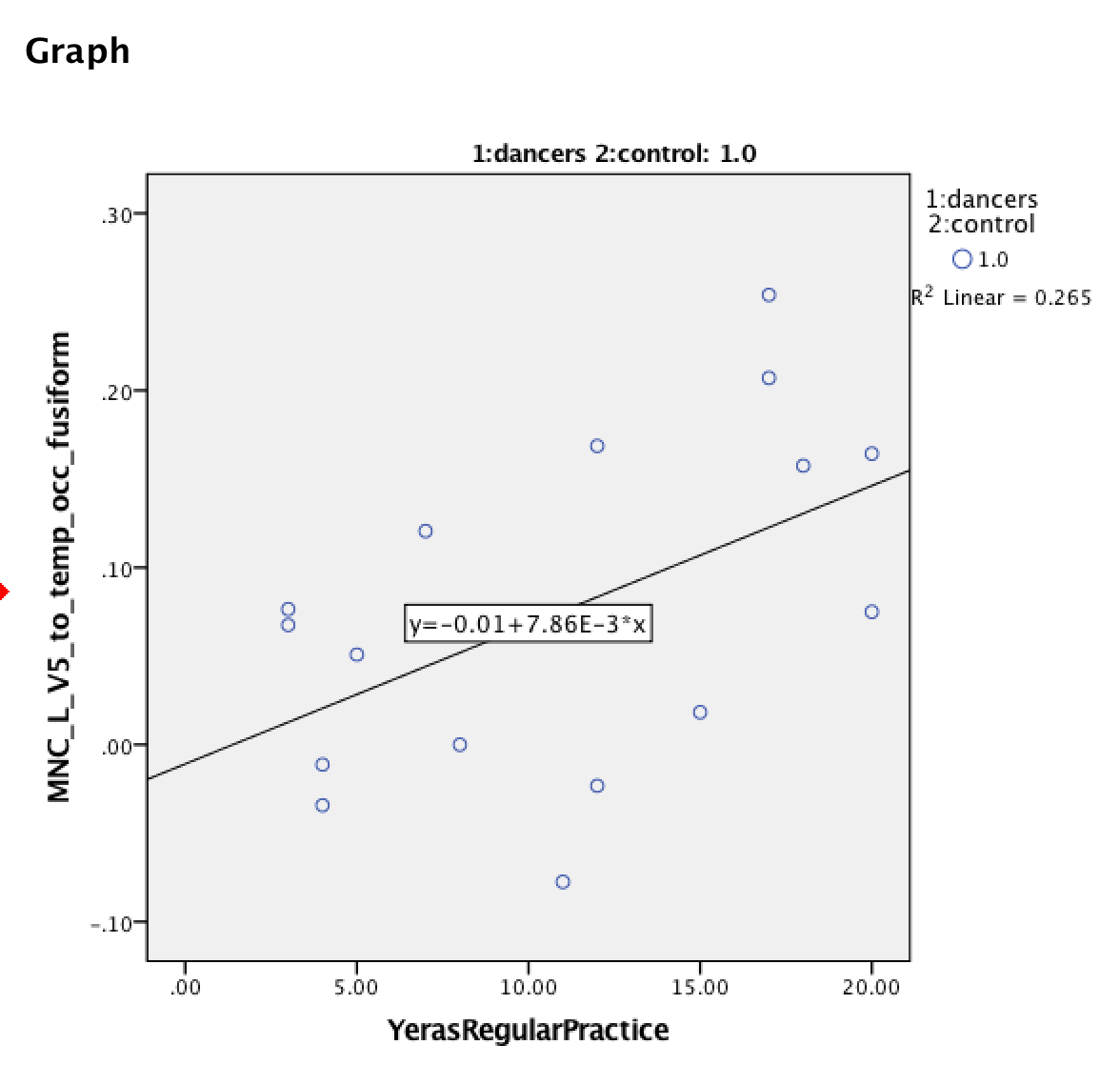


Scatterplot 4: Correlation between years of regular practice and FC between L V5 and temporal occipital fusiform cortex. (Dancers only)

**Supplementary materials**: References

Bennett G, Seashore H, Wesman A. 1997. Differential Aptitude Test. San Antonio: The Psychological Corporation.

Ekstrom R, French J, Harman H, Dermen D. 1976. Manual for Kit of Factor-referenced Cognitive Tests. Princeton: Educational Testing Service.

Raven J. 1962. Advanced Progressive Matrices: Sets 1 and 2. London: H. K. Lewis.

Salthouse TA, Babcock RL. 1991. Decomposing adult age differences in working memory. Dev Psychol. 27:763–776.

Wechsler D. 1997. Wechsler Adult Intelligence Scale. Third. ed. San Antonio: The Psychological Corporation.

Zachary RA. 1986. Shipley Institute of Living Scale: Revised Manual. Los Angeles: Western Psychological Services.
